# 4-Hydroxybutyrate (4HB) released from poly-4-hydroxybutyrate scaffolds does not impact hallmark phenotypes of cancer in malignant or non-malignant breast cells

**DOI:** 10.1186/s13058-026-02234-7

**Published:** 2026-02-14

**Authors:** Sakib F. Elahi, David P. Martin, Yong Wan, Li Zhang, Daniela J. Romero, Remya Kommeri, Madeline C. Cramer, Linsey Reavie, Adam C. Mercer, Diana Catalina Ardila, George S. Hussey, Stephen F. Badylak

**Affiliations:** 1 Becton, Dickinson and Company, 100 Crossings Blvd, Warwick, RI 02886 USA; 2 Reaction Biology, 1 Great Valley Parkway, Suite 2, Malvern, PA 19355 USA; 3 McGowan Institute for Regenerative Medicine, University of Pittsburgh, 450 Technology Dr., Pittsburgh, PA 15219 USA; 4 LBR Consulting LLC, 37030 N Conestoga Trail, Cave Creek, AZ 85331 USA

**Keywords:** Poly-4-hydroxybutyrate, *In vitro* cancer assay, Implantable absorbable scaffold, Breast reconstruction

## Abstract

**Background:**

Poly-4-hydroxybutyrate (P4HB) scaffolds are increasingly used to reinforce soft tissue during implant-based reconstruction after mastectomy. P4HB undergoes hydrolytic degradation to a natural metabolite, 4-hydroxybutyrate (4HB). Understanding the direct effects of 4HB on cancer cells is essential for assessing the oncologic safety of P4HB scaffolds used in breast reconstruction surgery. The aim of this study was to evaluate the effects of sodium 4-hydroxybutyrate (Na4HB) on multiple, relevant human breast cancer and epithelial cell types using a panel of well-established in vitro assays aligned with several hallmarks of cancer.

**Methods:**

First, the clinically relevant tissue concentration of 4HB was determined via a rabbit model to quantify 4HB in the peri-implant tissue of P4HB scaffolds. Second, human breast cell types, including non-malignant HMEC and MCF-10A, and cancerous MCF-7, BT-474 and MDA-MB-231, were exposed to Na4HB at up to 10X the clinically relevant tissue concentration. Cells were then evaluated for cancer related phenotypes: metabolic activity (MTT assay), proliferation (BrdU assay), migration (Scratch and Transwell assays), and colony formation (soft agar assays). Specific inhibitory control compounds for each assay were included to confirm assay performance.

**Results:**

The average peri-implant concentration of 4HB was found to be 163 µM after a simulated 1-year implantation in a rabbit model. Across the five assays and all three Na4HB concentrations, ranging from below to over 10X the peri-implant level (70, 350, 1750 µM), there was no statistically significant increase in any cancer-related phenotype including metabolic activity, proliferation, migration and colony formation in either malignant or non-malignant cell types compared to controls treated with either the PBS vehicle or sodium acetate as determined by one-way ANOVA followed by Tukey’s multiple comparison test (*p* > 0.05).

**Conclusions:**

This comprehensive, in vitro evaluation suggests that 4HB does not modify growth or activity of malignant or non-malignant breast cells at concentrations up to 10X the peri-implant level. While these findings suggest that 4HB released from degrading P4HB Scaffolds is unlikely to promote oncogenic behavior in vitro, further co-culture systems, in vivo studies and long-term clinical data can be used to further assess the oncologic safety in breast reconstruction.

**Supplementary Information:**

The online version contains supplementary material available at 10.1186/s13058-026-02234-7.

## Background

Breast reconstruction is a common procedure following mastectomy, with implant-based techniques being the predominant approach [1, 2]. These techniques are often complemented with biomaterials used for soft tissue reinforcement including porcine-, bovine-, and human-sourced acellular dermal matrices (ADMs) as well as fully absorbable meshes or biosynthetic scaffolds. Although no surgical mesh products are currently cleared or approved by the FDA specifically for use in breast surgery, ADMs are widely used in implant-based reconstructions [1, 3].

Fully absorbable scaffolds made from poly-4-hydroxybutyrate (P4HB) have emerged as cost-effective alternatives to ADMs due to their ease of handling in peri-pectoral planes, ability to stimulate vascularized collagenous tissue ingrowth, and degradation by 18 to 24 months [4, 5]. Recent studies have reported outcomes with P4HB scaffolds including reduced infection and seroma rates, shorter drain duration, and lower material costs compared to ADM in both subpectoral (dual plane) and prepectoral settings [4]. A four-year review of prepectoral reconstructions using P4HB scaffolds also noted high tissue integration and low capsular contracture rates, along with patient satisfaction [5]. Histopathological analysis of explanted specimens showed constructive host tissue response to P4HB scaffolds in breast surgery [6], and comparative data demonstrated that both synthetic and biologic scaffolds have no significant difference in capsular contracture rates, suggesting protective effects in high-risk settings [7]. These findings, while promising, are limited by retrospective study designs and patient selection, and long-term oncologic outcomes associated with P4HB scaffold use have not been extensively studied. Additionally, prospective studies are needed to validate these observations and assess long-term safety.

This is particularly relevant given that regenerative biomaterials are designed to promote tissue repair through wound-healing processes that are also co-opted during tumor progression. Wolf et al. highlighted the theoretical risks that pro-regenerative tissue environments might inadvertently support tumor growth, particularly when scaffolds are implanted near residual cancer cells following oncologic surgery [8]. While clinical experience has not shown evidence of scaffold-induced tumorigenesis, the lack of mechanistic data has prompted calls for rigorous preclinical evaluation.

As the clinical adoption of P4HB scaffolds expands, particularly in patients with a history of breast cancer, it is critical to evaluate the oncologic safety of these materials and their degradation products. P4HB degrades into 4HB, a naturally occurring metabolite present in many tissues [9], including the central nervous system (CNS), where 4HB has demonstrated protective effects against oxidative stress [10, 11]. 4HB is catabolized through succinic acid and the Krebs cycle [12] and so may impact energy production and metabolism of peri-implant tissue or nearby cancer cells. Studies have shown that 4HB upregulates antimicrobial peptides (AMPs) in macrophages [13], which have been explored as an emergent anticancer strategy [14]. Structurally, 4HB is closely related to ß-hydroxybutyrate (BHB), a ketone body known to influence cancer cell behavior in a context-dependent manner with both anti- and pro-tumorigenic properties. BHB has demonstrated antitumorigenic effects by impairing proliferation, migration, and stemness characteristics through HDAC4 inhibition [15]. However, evidence also suggests that elevated levels of BHB may promote tumor progression in certain contexts; for example, in colorectal cancer, BHB has been shown to accelerate proliferation and metastasis via ACAT1-mediated metabolic reprogramming [16]. Although the effect of 4HB on cancer cells has not been studied extensively, its biological similarity to BHB and its role in immune modulation suggest it may influence cancer cell behavior. To date, there is limited evidence to determine how 4HB may affect residual or dormant breast cancer cells in the peri-implant environment, underscoring the need for further research.

As P4HB scaffolds gain traction in breast reconstruction, particularly among patients with a history of breast cancer, it becomes increasingly important to understand how P4HB degradation products may interact with the surrounding tissue environment. While in vivo models have provided insights into the biocompatibility of P4HB scaffolds and their favorable host-tissue response [17, 18], there is a paucity of in vitro data evaluating the direct effects of absorbable materials on cancer cell behavior. This is especially relevant given the pro-regenerative environment in the tissue that surrounds a P4HB scaffold [19]. To address this gap, the present study evaluates the effects of sodium 4-hydroxybutyrate (Na4HB) on multiple, relevant human breast cancer cell lines and breast epithelial cells using a panel of well-established in vitro assays aligned with hallmarks of cancer such as metabolic activity, proliferation, migratory potential and colony formation [20–24]. To our knowledge, this is the first study to directly investigate the cellular effects of Na4HB in the context of breast cancer, providing mechanistic insights into its potential oncologic impact. These findings will inform the oncologic risk profile of P4HB Scaffold use in breast reconstruction, particularly for patients with a history of breast cancer.

## Methods

**Fig. 1:**
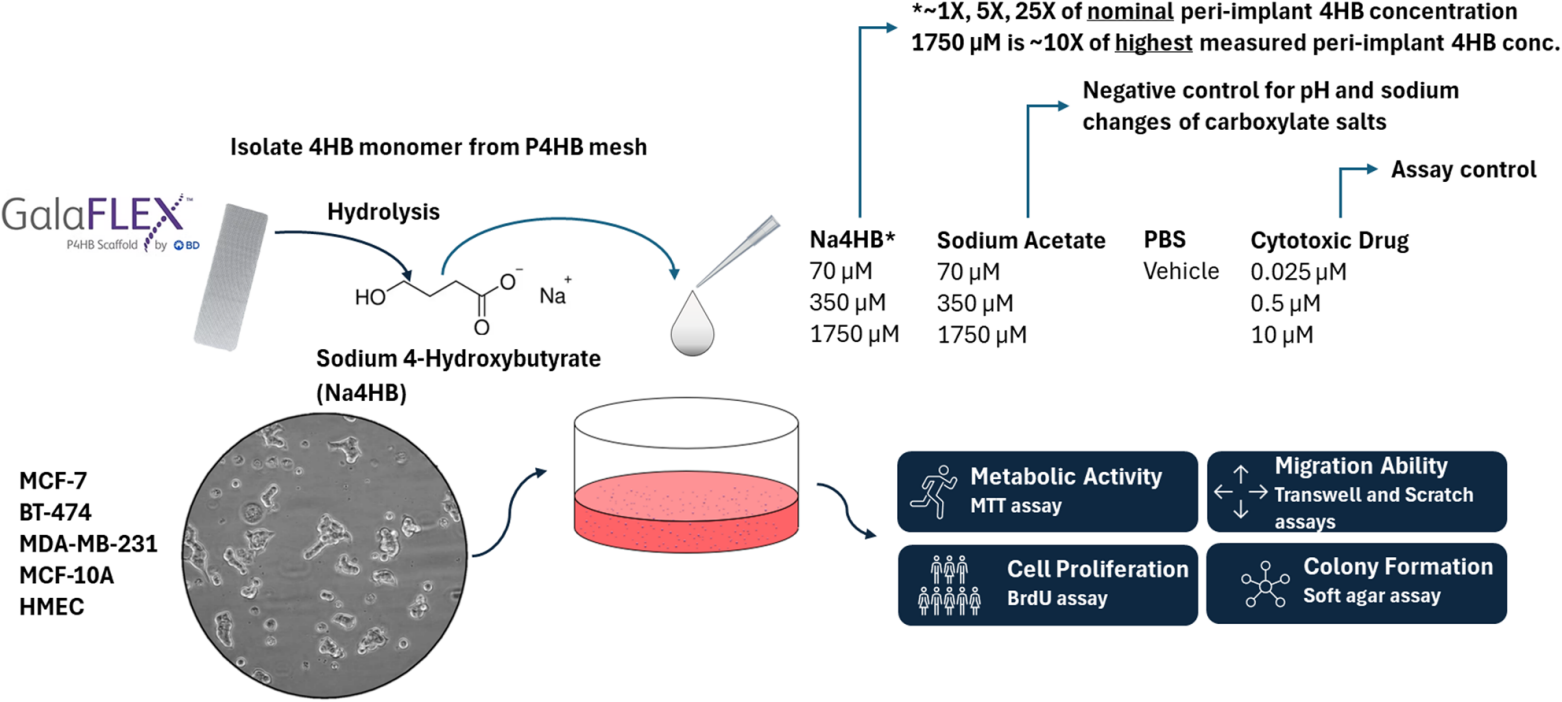
Schematic of study design. A series of in vitro assays were used to assess some hallmarks of cancer on human breast cancer or mammary epithelial cells when exposed to 4HB

A two-stage experimental program was undertaken. First, an in vivo study in rabbits was conducted to determine the peri-implant concentration of Na4HB in the tissue surrounding two different P4HB scaffolds. Second, Na4HB concentrations up to 10 times higher than those measured in peri-implant tissue in the rabbit model were used in a series of in vitro assays to assess some of the hallmarks of cancer in several breast cancer and epithelial cell types as shown in Fig. 1. The 10-fold upper dose is informed by regulatory body guidance on high-dose selection in preclinical studies [25]. Together, this approach provides a rigorous and clinically meaningful assessment of the oncologic safety of the monomeric degradation product of P4HB scaffolds. It was hypothesized that human breast cancer or epithelial cell types exposed to Na4HB will not exhibit a biologically meaningful increase in metabolic activity, proliferation, migration, or colony-forming ability compared to the PBS vehicle or the same dose of sodium acetate control.

### In vivo peri-implant concentration of 4HB in a rabbit model

All animal procedures were conducted in accordance with institutional guidelines and approved by the Institutional Animal Care and Use Committee (IACUC) under protocol number 23,790,053. A total of 12 New Zealand White rabbits weighing > 3.5 kg at time of implantation were included in the study. Each received four subcutaneous implants (5 × 5 cm) along the dorsum; two implants on each side of the spine. Each animal was implanted with one device type, either a P4HB scaffold (GalaFLEX™ Scaffold, BD, Franklin Lakes, NJ), a low Mw P4HB scaffold (pre-degraded GalaFLEX™ Scaffold), or a polypropylene control mesh (Bard™ Soft Mesh, BD, Franklin Lakes, NJ); with a total of 4 animals per device type. The pre-degraded GalaFLEX™ Scaffold represents the P4HB material at a later stage of degradation due to its reduced Mw. Bard™ Soft Mesh is made from polypropylene which is a non-degradable polymer that does not contain P4HB; for this reason, any residual 4HB in the peri-implant tissue surrounding this mesh would represent the concentration of native 4HB present in the tissue during the healing response. The mass of P4HB per sample was approximately 500 mg (areal density of 20 mg per sq. cm) for a total of approximately 2 g of implanted P4HB in the GalaFLEX ™ Scaffold animals. The sample size per time point for each group was eight. Three spare animals (one for each device type) were implanted to replace any animals excluded during the study; however, there were no animals excluded for either time point, and the spare animals were not included in the analysis.

**Fig. 2:**
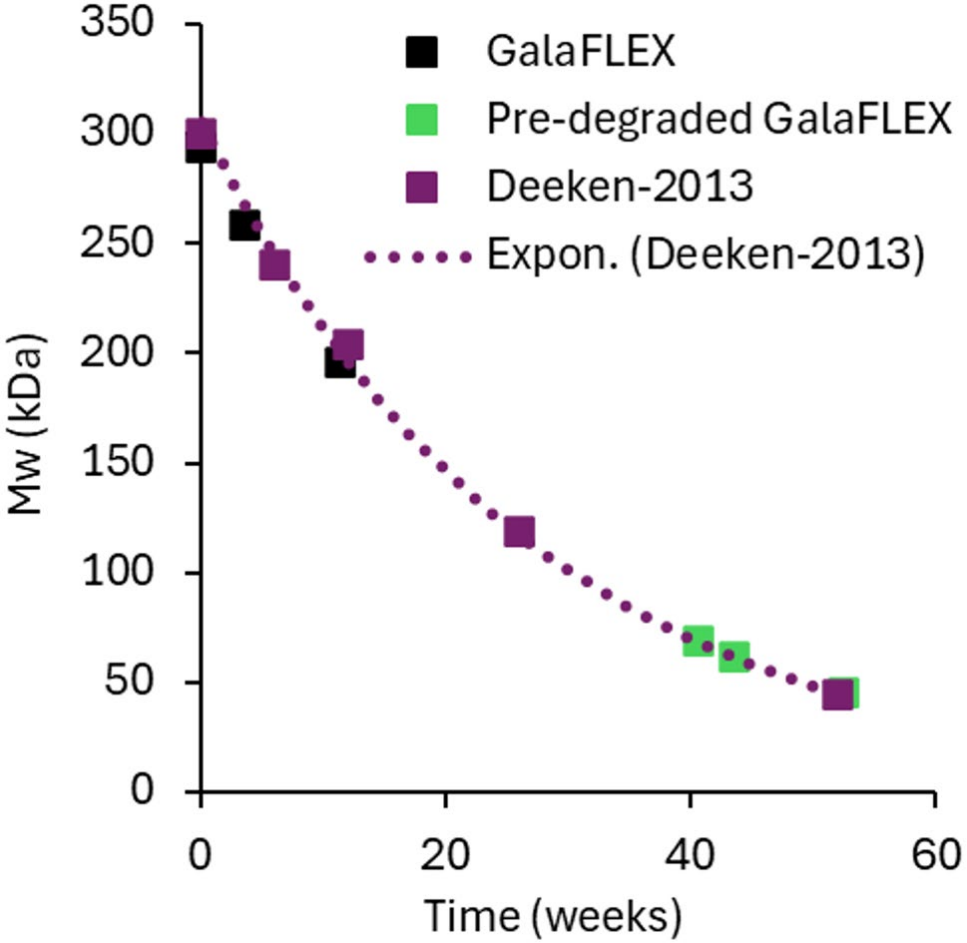
Mw values for GalaFLEX™ Scaffold (black, 0–12 weeks) and pre-degraded low Mw GalaFLEX™ Scaffold (green, 0–12 weeks) after rabbit implantation, superimposed on a typical P4HB scaffold Mw loss profile (purple) from a previous porcine implantation study [17]. Data supports that the implanted pre-degraded GalaFLEX™ at 4 and 12 weeks simulates 43 and 52 weeks of implantation. The dotted line shows the least squares best fit for the Deeken data, (y = 305.97e^-0.037x^, R^2^ = 0.9977) used to estimate simulated exposure time for the pre-degraded, low Mw P4HB scaffold

Animals were survived for 4 or 12 weeks, which represent a sub-chronic and a chronic timepoint that is twice the typical wound healing time of 6 weeks. Implantation of the pre-degraded P4HB scaffolds and comparison of their Mws to that from a previously reported study [17] allowed the simulation of longer timepoints, while minimizing the length of the animal experiment. The Mw loss curve of P4HB mesh in vivo correlates very well with implantation time and follows an exponential curve (see Fig. 2). Thus, the Mws of the predegraded scaffolds after 4 and 12 weeks in vivo were used to calculate simulated implantation timepoints based on the Mw vs. time plots from the previous study [17].

Animals were observed daily for incision site abnormalities and general animal health (attitude, appetite, urine/fecal output, and pain/distress). At the scheduled terminal time points, the rabbits were humanely euthanized followed by a limited necropsy to collect the implanted devices with surrounding tissue. The explanted devices/tissues were immediately frozen and shipped overnight to the testing lab.

The local concentration of 4HB in the tissue surrounding the devices was determined by Gas Chromatography (GC) assay after extraction of the 4HB from the explanted scaffold/tissue samples as described in the supplemental information. A total of 6 samples were used per scaffold type at each time point (*n* = 6, 3 samples per animal).

Separately, two explanted samples per time point (i.e. one per animal), were analyzed by Gel Permeation Chromatography (GPC) to determine the weight average molecular weight (Mw) of the residual polymer and to confirm agreement of the Mw loss profile of the P4HB devices in the present study with previous results. The Mw was determined after the P4HB-containing samples were digested with collagenase (Type I, from Clostridium histolyticum) to remove ingrown tissue. The recovered P4HB was dissolved in chloroform, and tested for Mw by GPC against polystyrene standards of low polydispersity and known Mw. Details on the methodology for measuring the 4HB local concentration, and characterization via GC and GPC including chromatographic parameters are described in the Supplementary Materials.

### In vitro cancer cell assays

#### Preparation and characterization of Na4HB monomer from GalaFLEX™ Scaffold

To degrade P4HB into its 4HB monomer, the GalaFLEX™ Scaffold was hydrolyzed in 3 M hydrochloric acid (HCl) at 37 °C over 9 days. After complete dissolution and hydrolysis of the P4HB, the solution was neutralized with sodium hydroxide to produce the sodium salt of 4HB (i.e., sodium 4-hydroxybutyrate, Na4HB) and sodium chloride. The solution was lyophilized to a powder and the Na4HB was isolated and desalted by ethanol extraction. The purity of the isolated Na4HB was determined analytically by Gas Chromatography after butanolysis. Additional analyses were performed including GC-MS, FTIR and ^1^H-NMR for chemical identity, Ion Selective Electrode quantification for sodium and chloride content and Karl Fischer titration for residual water. See Supplemental information.

The acid form of 4-hydroxybutyrate is not stable in aqueous solution, since it lactonizes to form the cyclic lactone gamma-butyrolactone. At physiological pH, 4HB ionizes nearly completely to its conjugate base, and so the carboxylic acid salt was chosen as the most relevant and convenient form of 4HB for these assays. Sodium was chosen to prepare the salt as it is the most prevalent cation in the body and would be the form of the monomer that would most likely be present at physiological pH although it is expected to undergo rapid exchange with other positively charged ions that are available in vitro or in vivo. The impact of the sodium counter ion is controlled in the in vitro assays by including sodium acetate as a benign control at the equivalent concentrations as Na4HB.

#### Cell line selection and culture conditions

The human breast cancer MCF-7, BT-474, and MDA-MB-231 cell lines, human breast epithelial MCF-10A and HMEC cells, and their culture media were purchased from American Type Culture Collection (ATCC). The selection of human breast cancer cell lines was predicated upon the cell types commonly observed within invasive breast cancers such as Invasive Ductal Carcinomas (IDC). Conversely, human breast epithelial cells were chosen for their representation of the non-cancerous state in both an immortalized and endogenous replicative capacity. Once received, cells were thawed, expanded, and frozen to maintain a low passage number for each experiment. MCF-7 cells were cultured in EMEM + 10% FBS + 10 µg/mL insulin. BT-474 cells were cultured in Hybri-Care medium plus 1.5 g/L sodium bicarbonate + 10% FBS. MDA-MB-231 cells were cultured in DMEM + 10% FBS. MCF-10A cells were cultured in DMEM: F12 medium + 5% HS + 20 ng/mL EGF + 5 µg/mL hydrocortisone + 100 ng/mL cholera toxin + 10 µg/mL insulin. HMECs were cultured in Mammary Epithelial Cell Basal Medium + Mammary Epithelial Cell Growth Kit. Cells were incubated at 37 °C in a humidified atmosphere containing 5% CO_2_. Details on culture media, chemicals and reagents used for the in vitro study including catalogue numbers are found in Supplemental information. All in vitro assays were performed in sextuplicate on three separate days.

#### Effect of Na4HB on cell metabolic activity

The impact of Na4HB on cell metabolic activity was measured via the MTT colorimetric assay [20]. MCF-7, BT-474, MDA-MB-231, MCF-10A, or HMEC cells were seeded (1000 cells/ well) in 25 µL of complete culture media in 384-well plates and incubated for 20–24 h. The media was replaced with fresh medium containing compound, either Na4HB (0, 70, 350 or 1,750 µM), sodium acetate (0, 70, 350 or 1,750 µM), or staurosporine (0.025, 0.5 or 10 µM). The metabolic activity was monitored after 72 h using CellTiter 96^®^ Non-Radioactive Cell Proliferation Assay (MTT) from Promega following the manufacturer’s instructions. Absorbance was measured at a wavelength of 490 nm using the Envision 2104 Multilabel Reader (Perkin Elmer, Santa Clara, CA). Comparison was made to a PBS vehicle control. 2X cell number served as a positive control to demonstrate headroom in the assay. Results were reported as percent cell metabolic activity relative to PBS vehicle control.

#### Effect of Na4HB on cell proliferation

The impact of Na4HB on cell proliferation was measured via the BrdU ELISA assay [21]. BT-474 and MDA-MB-231 cells (500 cells/well) and MCF7, MCF-10A and HMEC (1000 cells/well) were seeded in 100 µL of culture media in 96-well plates and incubated at 37 °C 5% CO_2_ for 20–24 h. The media was replaced with fresh medium containing compound, either Na4HB (0, 70, 350 or 1,750 µM), sodium acetate (0, 70, 350 or 1,750 µM), or staurosporine (0.025 µM, 0.5 µM or 10 µM), and cells were incubated for 24 h (MDA-MB-231), 48 h (MCF-7 and BT-474), or 72 h (MCF-10A and HMEC). After incubation, proliferation was measured using the BrdU Cell Proliferation Assay Kit (Sigma-Aldrich) following the manufacturer’s instructions. Absorbance was measured at a wavelength of 460 nm using the Envision 2104 Multilabel Reader (Perkin Elmer, Santa Clara, CA). Comparison was made to a PBS vehicle control. 2X cell number served as a positive control to demonstrate headroom in the assay. Results were reported as % BrdU incorporation relative to PBS vehicle control.

#### Effect of Na4HB on cell migration

To assess the effect of Na4HB on cell migration of breast cancer cell and mammary epithelial cell lines, Transwell and Scratch assays were performed. The Transwell assay was employed to assess the effects of Na4HB on migration characteristics of BT-474, MDA-MB-231, MCF-10A, and HMEC cells. Migration characteristics of MCF-7 cells were evaluated in a Scratch assay as this cell line exhibits a low migratory phenotype in the Transwell assay [26]. Details on the experimental design for the Transwell and Scratch assays are described below.

##### Effect of Na4HB on Transwell assay to measure cell migration

The Transwell migration assay was performed for BT-474, MDA-MB-231, MCF-10A, and HMEC cells using IncuCyte^®^ Live-Cell Analysis System. Each insert well of the IncuCyte^®^ Clearview 96-Well Plates was pre-coated with 150 µL of collagen IV solution (5 µg/mL) prior to cell seeding. Coating was not required for MDA-MB-231. Briefly, 60 µL of base media containing either 5000 cells (HMEC), 2500 cells (for BT-474 and MCF-10A) or 1000 cells (for MDA-MB-231) and the compounds of interest (PBS, Na4HB or sodium acetate at 0, 70, 350 or 1750 µM or bosutinib at 0.025 µM, 0.5 µM or 10 µM) were added to the IncuCyte^®^ Clearview insert wells. The cells were incubated for 15 min at room temperature on a level surface. 200 µL of the complete culture media containing the compounds of interest (PBS, Na4HB or sodium acetate at 0, 70, 350 or 1750 µM or bosutinib at 0.025, 0.5 or 10 µM) were added to each of the reservoir wells. Reservoir wells used for MDA-MB-231 cells were supplemented with 10 ng/mL EGF as a chemoattractant. The insert was transferred to the pre-loaded reservoir plate and cell migration was monitored in the IncuCyte^®^ S3 Live-Cell Analysis System at 37 °C, 5% CO_2_ for 96 h after a 60-minute warm up time. The phase contrasted object count was measured at regular intervals for the entirety of the predetermined migration period. Results were reported as total phase contrast object count as measured from the bottom of the Transwell membrane.

##### Effect of Na4HB on scratch assay to measure gap closure

To evaluate the impact of Na4HB on cell migration of MCF-7 cells, the scratch wound healing assay was used [23], as the Transwell method described above was not suitable for MCF-7 cell line. MCF-7 cells were seeded into collagen IV-coated 96-well IncuCyte^®^ ImageLock Plates in 100 µL of complete media containing 5 × 10^4^ cells. The plates were incubated at 37 °C, 5% CO_2_ for 20–24 h. Standardized wounds were simultaneously created in all wells using the IncuCyte^®^ Woundmaker Tool. Debris and non-attached cells were removed by gently washing the wells twice with complete media. After washing, 180 µL of media was added back to each well and the compounds of interest were added (PBS, Na4HB or sodium acetate at 0, 70, 350 or 1750 µM) to a total volume of 200 µL. Dasatinib (0.025, 0.5 or 10 µM) was used as an anti-migration control to assess in vitro system suitability. The plates were incubated in the IncuCyte S3 instrument at 37 °C, 5% CO₂ and wound closure was recorded in real time every 12 h for 96 h. The percentage of wound confluence relative to the starting time point (0 h) was measured, and results were reported as relative gap closure (%).

#### Effect of Na4HB on anchorage-independent cell growth and colony formation

To assay anchorage-independent cell growth and colony formation, a soft agar colony formation assay was performed [24]. Cells were suspended in 150 µL of 0.4% agar in complete media on top of a bottom agar layer of 62.5 µL 0.6% agar in complete media within 96-well plates. The number of cells per well was 750 for BT-474 and MCF-10A, 1000 for MCF-7, MDA-MB-231, and HMEC. Complete media supplemented with the different treatment conditions (Na4HB, sodium acetate, staurosporine or no treatment (PBS)) was added. The final concentrations of Na4HB or sodium acetate (SA) were 70 µM, 350 µM and 1750 µM while the final concentrations of staurosporine were 0.025 µM, 0.5 µM and 10 µM. The plates were sealed with sterile porous cell culture film to prevent the soft agar from drying out and the plates were incubated at 37 °C, 5% CO_2_ for 14 days.

The plates were checked twice each week for colony growth and the color of the complete media (to ensure nutrients were not depleted). After 1 week, 60% of the average volume of remaining media was removed from each well and replaced with an equal volume of fresh complete media containing the compounds of interest. On the day of plate scanning, the culture media was aspirated carefully, 50 µL MTT Dye Solution was added to each well to aid visualization of the colonies and the cells were incubated at 37 °C in CO_2_ incubator for an additional 2 h to complete staining. Before scanning, each well was filled with PBS to reduce optical effects of the liquid meniscus, and the plates were scanned using the GelCount (Oxford Optronix) instrument. The cells in some wells underwent clonal outgrowth on the bottom surface of the plate, which was not anchorage-independent (i.e., did not represent colonies). This interfered with the software’s ability to count colonies accurately. Therefore, these wells were deemed invalid and were not included in the colony counting data. Outgrowths occurred in each treatment case (Na4HB, sodium acetate of PBS) and at each concentration and so were not dependent on the treatment compounds tested. To ensure enough data points for analysis, we increased the number of technical wells per assay day/biological replicate. For each cell line, the final number of replicates was determined by the condition with the fewest valid wells, known as the “n-value,” ensuring equal replicates per condition. Wells included in the analysis were selected sequentially by biological replicate, starting from the first valid well in each condition. The average number of colonies formed was calculated for each condition to enable comparison between treatments. At least eleven measurable wells across three biological replicates were achieved for each cell type. The number and size of colonies were determined in the valid wells using the GelCount™ Mammalian Cell Colony Counter (Oxford Optronix) and compared between groups. The number of colonies was determined as the cumulative number of objects with diameter between 50 and 300 μm.

### Statistics

For each experiment, data normality and equal variance was confirmed before proceeding with statistical tests. The mean for each assay day was calculated for each cell line (*n* = 3) and used for statistical analysis using Minitab software. One-way ANOVA and Tukey comparison tests were completed to evaluate the relationship between Na4HB and sodium acetate treatment at different concentrations or PBS vehicle control for each cell line. Data were presented as a mean +/- SEM. A p-value of ≤ 0.05 was considered significant.

## Results

### In vivo peri-implant tissue concentration of 4HB in a rabbit model

Peri-implant concentration of 4HB was measured after implantation of two different types of P4HB scaffolds in a rabbit model. Implantations were successfully performed and all animals survived to their designated terminal time points. Table 2 presents the GC results for the local concentrations of 4HB in the explanted samples at the 4- and 12-week time points.

**Table 2:** Concentrations of extracted 4HB from explanted samples at 4- and 12-weeks post implantation as analytically determined (µg/g wet tissue) and calculated for tissue conc. (µM)^α^

GalaFLEX™ Scaffold	4-weeks (*n* = 6)^†^	12-weeks (*n* = 6) ^†^
	13.5 (± 17.8) µg/g 107.4 (± 141.0) µM	8.6 (± 1.4) µg/g 68.5 (± 11.0) µM
Pre-degraded GalaFLEX™ Scaffold	11.8 (± 1.9) µg/g* 93.5 (± 15.5) µM*	20.5 (± 5.8) µg/g** 162.7 (± 46.0) µM**
Bard™ Soft Mesh	2.3 (± 1.4) µg/g 18.0 (± 11.0) µM	7.2 (± 1.5) µg/g 57.2 (± 12.2) µM

^†^*n* = 6 samples per scaffold type per time point, derived from 3 samples per animal x 2 animals. Technical replicates were processed independently within each biological replicate

* 43-week equivalent and ** 52-week equivalent based on previous data correlating Mw with implantation time. ^α^Concentrations in µM were determined by assuming the tissue is composed mostly of water and using the molar mass of 126 g/mol for Na4HB, ex. 13.5 µg/g translates to 13.5 mg/L or 107 µM

Separate samples of P4HB meshes were explanted and the residual polymer was prepared for molecular weight analysis by GPC to determine the extent of polymer degradation. The molecular weight of the explanted mesh samples was 258 kDa and 196 kDa for GalaFLEX™ and 62 kDa and 45k Da for pre-degraded GalaFLEX™ from the 4-week and 12-week time points, respectively. Figure 2 shows the Mw values for the GalaFLEX™ samples vs. time as compared to a historical plot of GalaFLEX™ Mw over time from previous published data. The data for the 4- and 12-week GalaFLEX™ samples fit the previously published Mw loss plot as expected [17]. The Mw values for the pre-degraded GalaFLEX™ samples after 4 and 12 weeks in vivo fall later on the curve and are representative of GalaFLEX™ Scaffold after approximately 43 and 52 weeks in vivo, respectively. Thus, in combination, the standard and pre-degraded GalaFLEX™ Scaffolds are representative of P4HB scaffolds that have been implanted for 4, 12, 43 and 52 weeks during which the scaffold would lose 85% of its original Mw and 20–30% of its original mass (Data not shown).

### In vitro cancer cell assays

All quantitative results are presented as mean ± SEM, normalized to PBS control, with *n* = 3 biological replicates per condition, as detailed in the Methods. For most assays, data are normalized to PBS control; migration object counts and colony counts are shown as absolute values, while Scratch assay time profiles are shown as percentage gap closure relative to time zero.

#### Characterization of 4HB isolated from a P4HB scaffold and used in the in vitro assays

The Na4HB product isolated from the GalaFLEX™ Scaffold for this work was of high purity as measured by GC analysis (96.2 wt%, 51% yield). Its chemical identity was confirmed by FTIR, ^1^H-NMR and GC-MS. ^1^H-NMR analysis also confirmed the lack of oligomeric or lactone contaminants. The expected presence of small amounts of chloride (2.6 wt %) and residual water (1.7 wt %) were quantified by Ion Selective Electrode and Karl Fischer Titration analyses and were the only identified contaminants in the preparation. See Supplemental Information for details.

#### 4HB does not increase metabolic activity or cell viability of human breast cancer or mammary epithelial cells

The metabolic activity of human breast cancer and mammary epithelial cell types after exposure to different concentrations of Na4HB was measured using an MTT assay as illustrated in the schematics of the study design (Fig. 1).

**Fig. 3:**
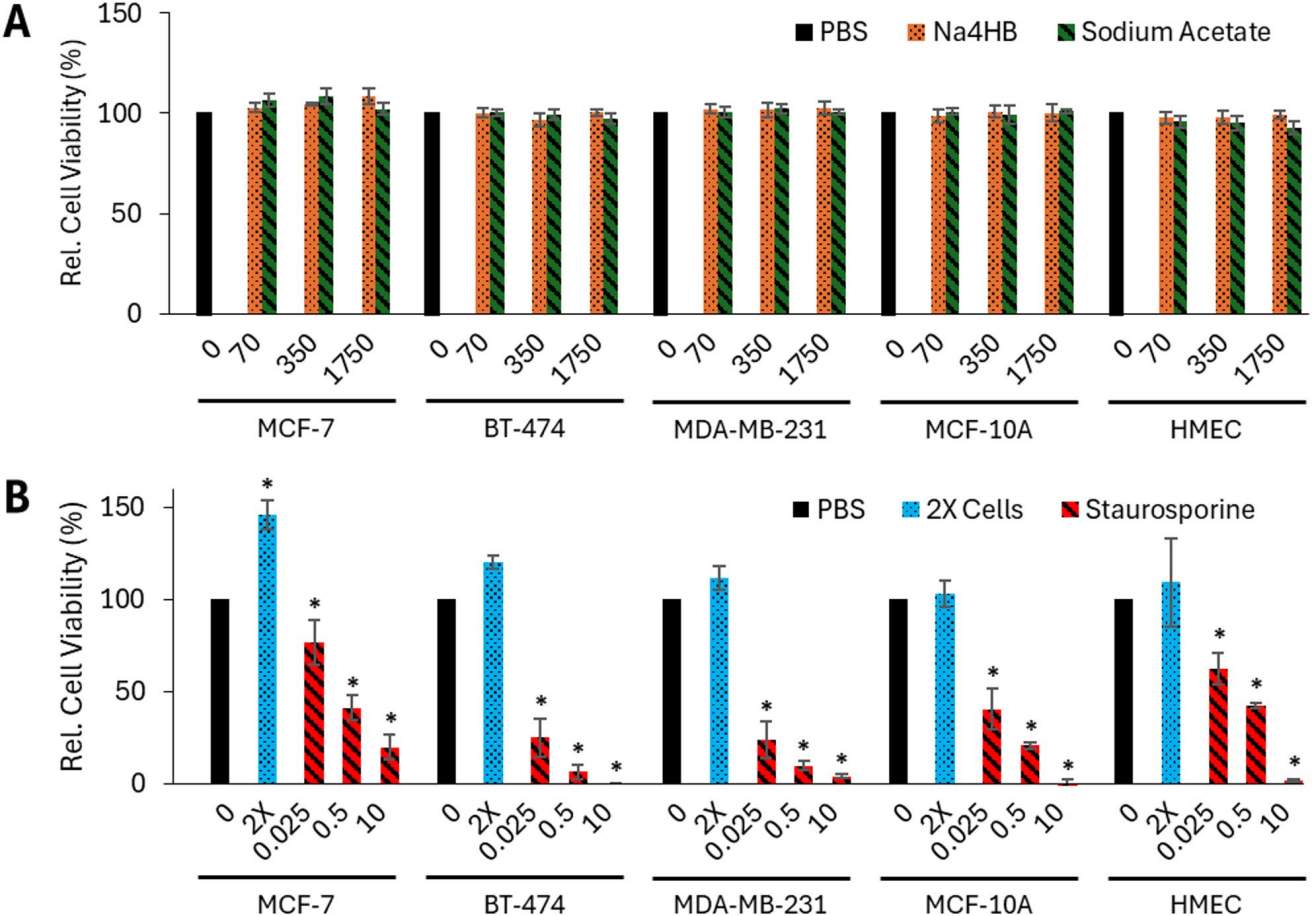
Cell viability of MCF-7, BT-474 MDA-MB-231, MCF-10A, and HMEC cells relative to PBS after 72 h exposure to **A** 0, 70, 350 or 1750 µM of Na4HB or sodium acetate; or **B** 0.025, 0.5 or 10 µM of staurosporine, or 2X cell dose (positive control), measured by MTT assay. Data represents the average response ± standard error of the mean for the three biological replicates. *p < 0.05 to PBS. No significant differences were found for Na4HB vs. PBS or Na4HB vs. sodium acetate for any of the groups

No significant differences in the metabolic activity of MCF-7, BT-474, and MDA-MB-231 breast cancer cells or MCF-10A and HMEC breast epithelial cells were observed between Na4HB and sodium acetate or PBS at any concentration through the MTT assay (Fig. 3A). This demonstrates that Na4HB concentrations up to 10 times higher than the peri-implant level does not increase metabolic activity of the human breast cancer or mammary epithelial cell types tested.

As shown in Fig. 3B, the 2X cell number positive control showed an increase in measured MTT concentration for the cancer cell lines, demonstrating the assay’s ability to measure an increase in metabolic activity or viability. In contrast, the inhibitory compound staurosporine demonstrated dose-dependent inhibition of cell viability across all five cell types. These controls demonstrated the ability of the assay to detect an increase or decrease in cell viability. As such, the assay was suitable for its intended purpose. Details on the statistical analysis results including p-values and confidence intervals can be found in the supplemental material as Excel tables.

#### 4HB does not increase the proliferation of human breast cancer or mammary epithelial cells

The proliferative activity of human breast cancer and mammary epithelial cell types after exposure to different concentrations of Na4HB was measured using a BrdU assay as illustrated in the schematics of the study design (Fig. 1).

**Fig. 4:**
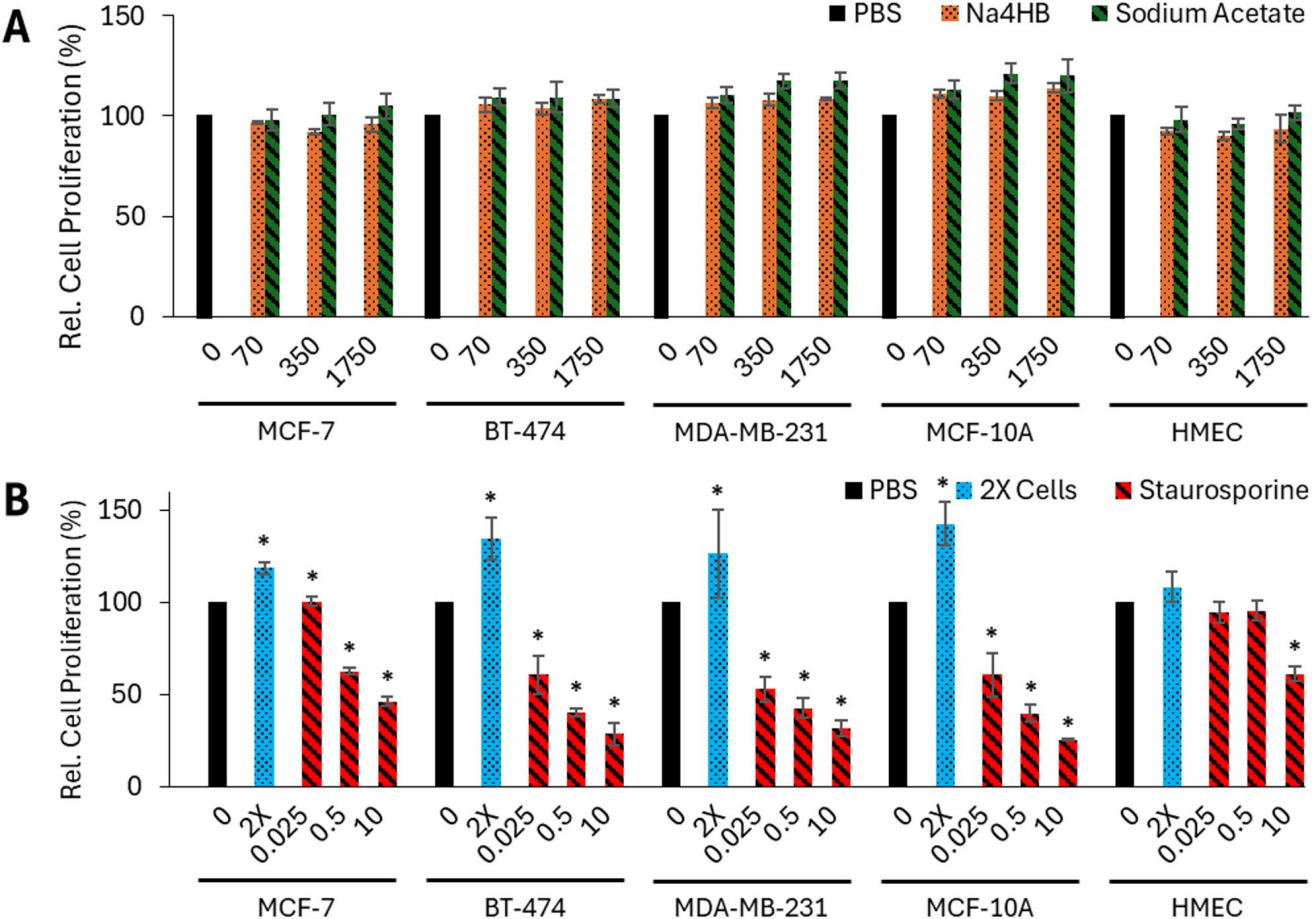
Cell proliferation of MCF-7, BT-474 MDA-MB-231, MCF-10A, and HMEC cells relative to PBS after 48 h exposure to **A** 0, 70, 350 or 1750 µM Na4HB or sodium acetate; or **B** 0.025, 0.5 or 10 µM of staurosporine, or 2X cell dose (positive control), measured by BrdU assay. Data represents the average response ± standard error of the mean for the three biological replicates. *p < 0.05 to PBS. No significant differences were found for Na4HB vs. PBS or Na4HB vs. sodium acetate for any of the groups

No significant differences in cell proliferation rate of MCF-7, BT-474, and MDA-MB-231 breast cancer cells or MCF-10A and HMEC breast epithelial cells were observed between Na4HB and sodium acetate or PBS at any concentration (Fig. 4A). This demonstrates that Na4HB concentrations up to 10 times higher than the peri-implant level does not increase the proliferation of the human breast cancer or mammary epithelial cell types tested.

As shown in Fig. 4B, the 2X cell number positive control showed an increase in the measured BrdU incorporation rate, demonstrating the assay’s ability to measure an increase in cell proliferation. In contrast, the reference compound staurosporine demonstrated a dose-dependent inhibition of cell proliferation across all five cell types. These controls demonstrate the ability of the assay to detect an increase or decrease in cell proliferation by rate of BrdU incorporation. Details on the statistical analysis results including p-values and confidence intervals can be found in the supplemental material as Excel tables.

#### 4HB does not increase the migratory potential of human breast cancer or mammary epithelial cells

The impact of varying concentrations of Na4HB on the migration of human breast cancer and mammary epithelial cell types was assessed using Transwell and Scratch assays, as illustrated in the schematics of the study design (Fig. 1). The Transwell assay was used for the BT-474, MDA-MB-231, MCF-10A and HMEC cells. The Scratch assay was used for the MCF-7 cells as the Transwell assay is not suitable for this cell line.

##### Transwell assay results

**Fig. 5:**
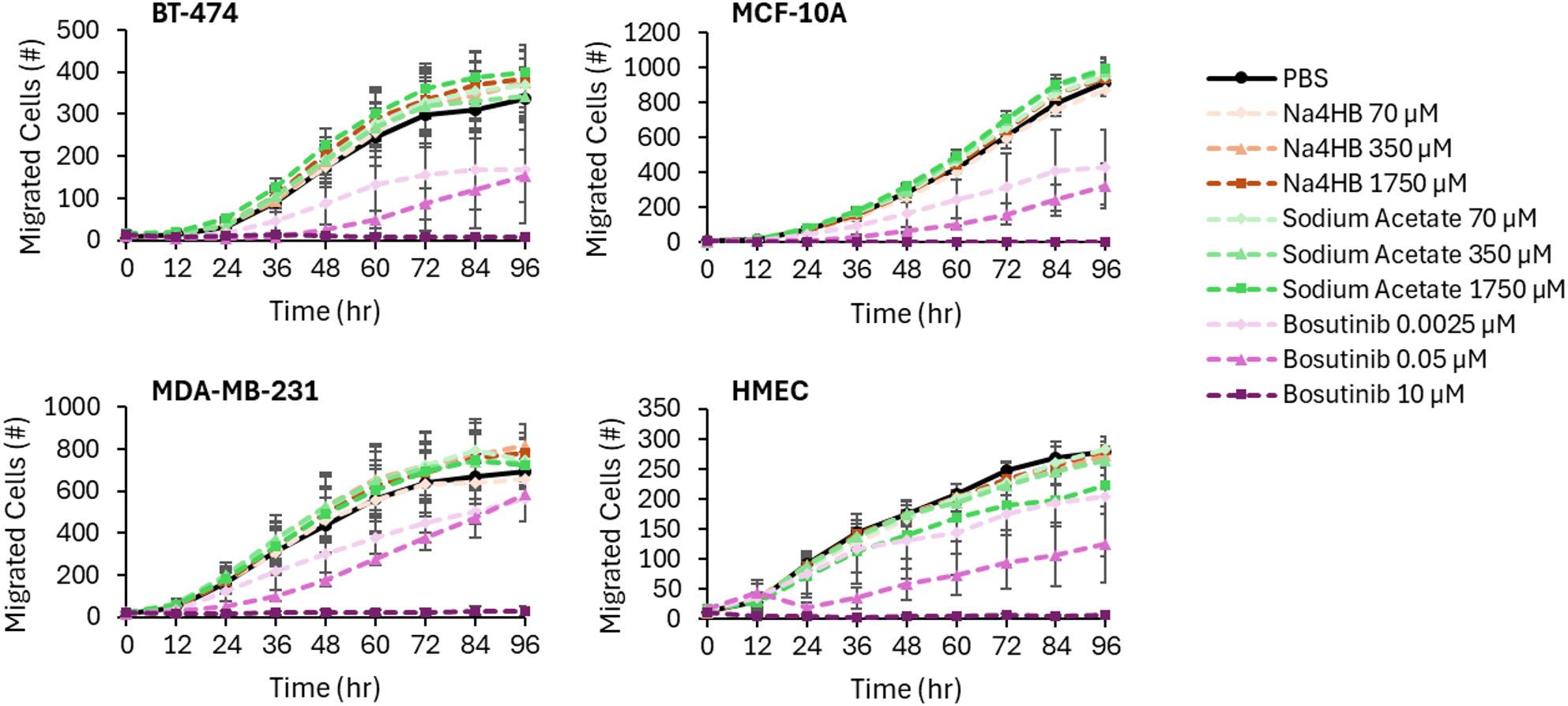
Transwell migration object cell counts over time for cancer and epithelial cells after exposure to PBS, Na4HB, sodium acetate, or bosutinib at the indicated concentrations. Data represent the average response ± standard error of the mean for the three biological replicates

The Incucyte equipment imaged the bottom of each well every 12 h so that the number of migrated cells could be monitored over the 96-hour experiment. An increase in migratory cells over time was observed for each cell type (Fig. 5). In each experiment, PBS, a vehicle control, was used to determine baseline migration for each cell type. A reference compound, bosutinib, demonstrated a dose-dependent inhibition of cell migration over time across the four cell types. Monitoring each well over time and the use of an inhibitory control enabled the assay to detect an increase or decrease in cell migration. As such, the assay was suitable for its intended purpose.

**Fig. 6:**
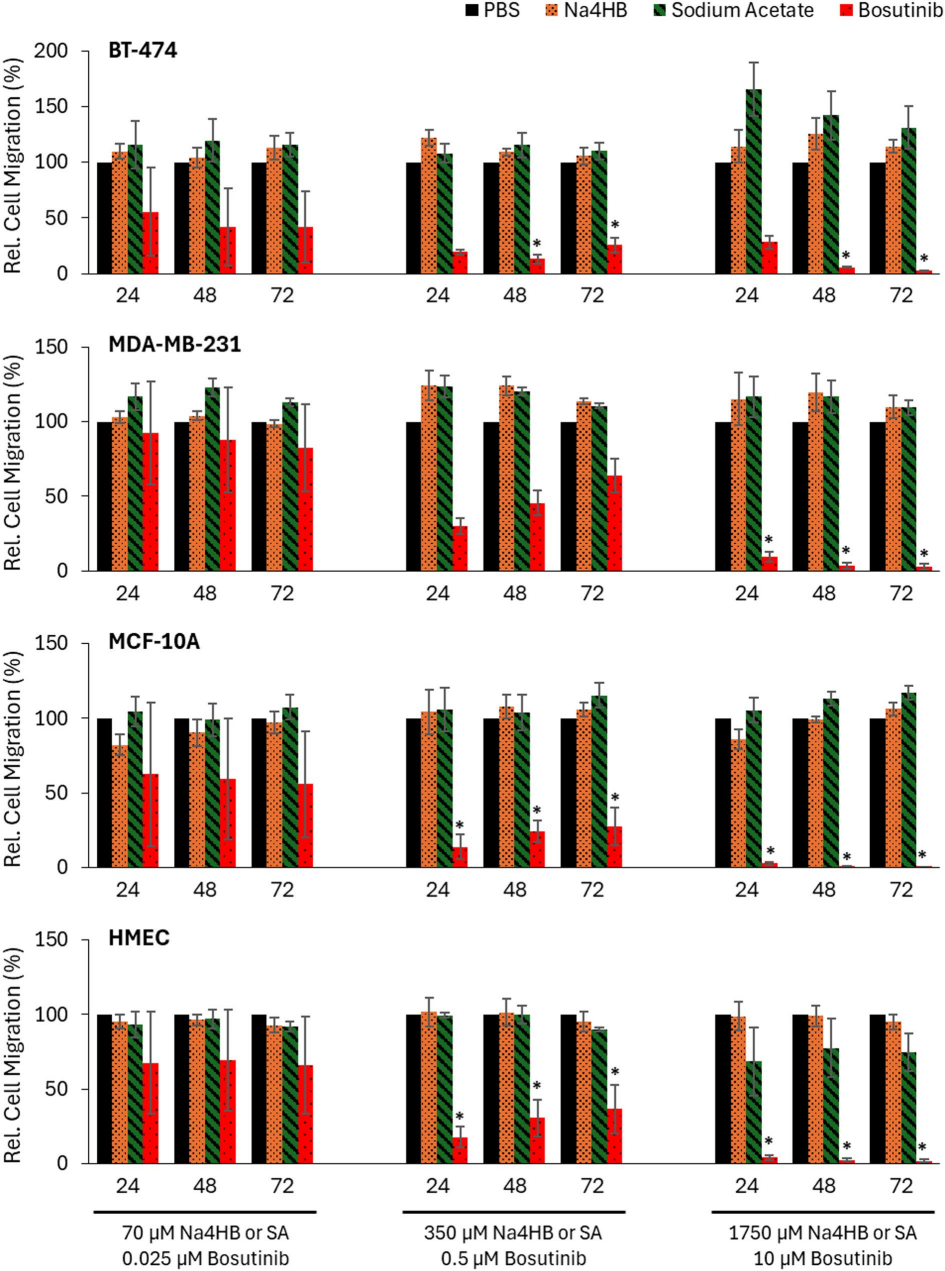
Transwell migration object cell counts (relative to PBS) at 24, 48, or 72 h for BT-474 and MDA-MB-231 cancer cells and MCF-10A and HMEC epithelial cells after exposure to PBS, Na4HB, sodium acetate or bosutinib at the indicated concentrations. Data represent the average response ± standard error of the mean for the three biological replicates. *p < 0.05 to PBS. No significant differences were found for Na4HB vs. PBS or Na4HB vs. sodium acetate for any of the groups

To enable a quantitative comparison between Na4HB and sodium acetate after treatment, the percentages of migratory cells (relative to PBS) were determined at each time point. Statistical comparisons were made spanning the linear portion of the assay, which was 24 to 72 h. Before 24 h, cells must settle onto the membrane and therefore object counts are prone to noise. After 72 h, cell migration can become exhausted due to complete cell migration, chemoattractant depletion, or loss of cell viability, etc. No significant differences in the percent of migratory cells for BT-474 and MDA-MB-231 breast cancer cells or MCF-10A and HMEC breast epithelial cells were observed between Na4HB and sodium acetate or PBS at any concentration. Summary bar charts for 24-, 48-, and 72-hour time points were selected as representative of the linear portion of the migration curve, to graphically present the means and standard errors of the mean for each cell type in Fig. 6. Details on the statistical analysis results including p-values and confidence intervals can be found in the supplemental material as Excel tables.

##### Scratch assay results

**Fig. 7:**
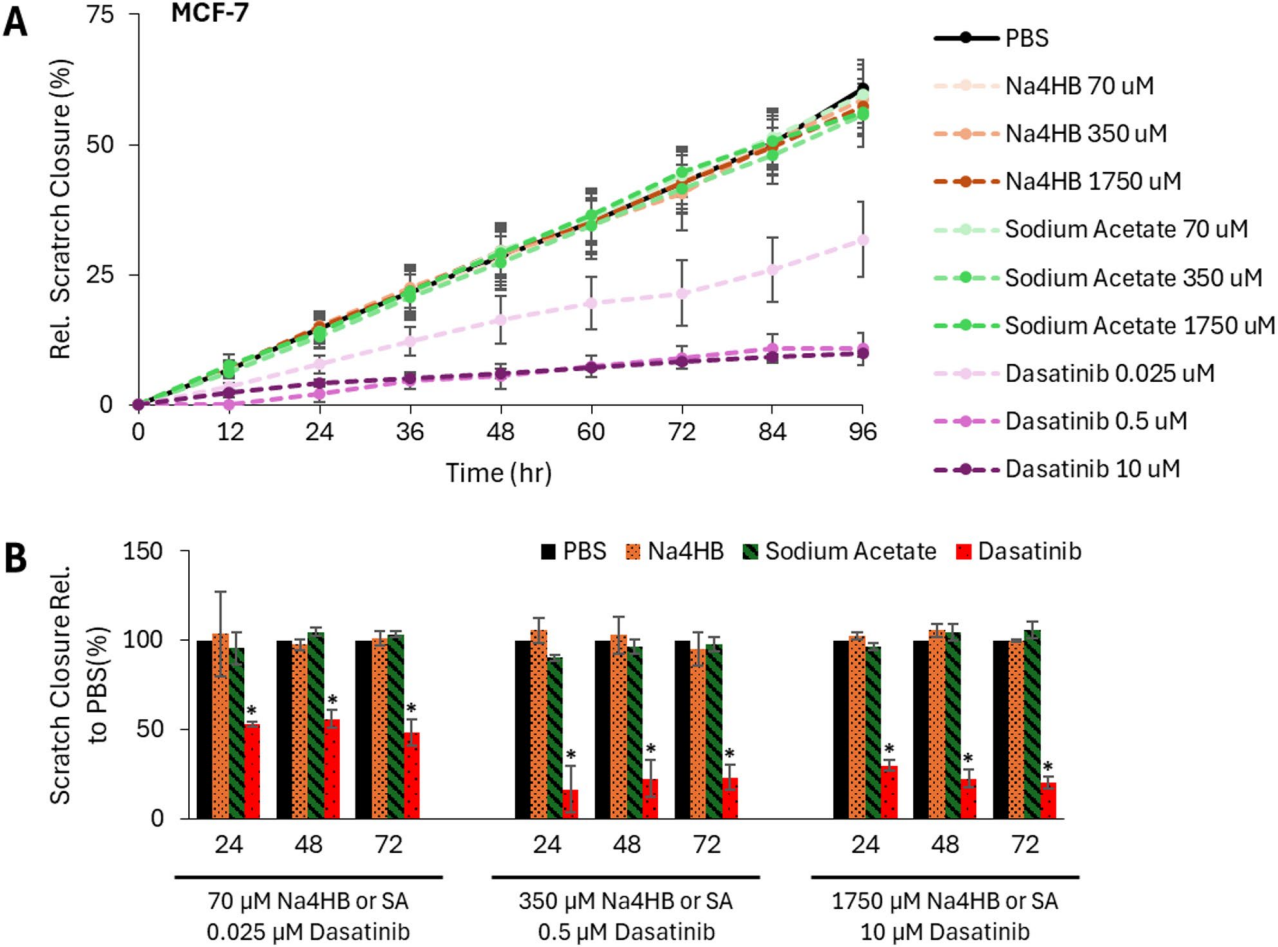
**A** Scratch wound gap closure for MCF-7 cells after exposure to PBS, Na4HB, sodium acetate, or dasatinib at the indicated concentrations. **B** Percentage of gap closure (relative to PBS) for at 24, 48, or 72 h. Data represent the average response ± standard error of the mean for the three biological replicates. *p < 0.05 to PBS. No significant differences were found for Na4HB vs. PBS or Na4HB vs. sodium acetate for any of the groups

Cell migration and gap closure by MCF-7 cells over time was observed (Fig. 7A). In each experiment, PBS, a vehicle control, was used to determine the baseline gap closure (i.e. migration) for MCF-7 cells. A reference compound, Dasatinib, demonstrated a dose-dependent inhibition of gap closure by MCF-7 cells over time. The continuous optical monitoring enabled the assay to detect the progression of gap closure over time and an increase or decrease in gap closure rate. As such, the assay was suitable for its intended purpose.

To enable a quantitative comparison between Na4HB and sodium acetate after treatment, the percentages of gap closure (relative to PBS) were determined at each time point. No significant differences in the percent of gap closure (i.e. migration) for MCF-7 cells were observed between Na4HB and sodium acetate or PBS at any concentration. Summary bar charts for 24-, 48- and 72-hours were selected as representing the linear portion of the curve, to graphically present the means and standard errors of the mean. The data are shown in Fig. 7B. Details on the statistical analysis results including p-values and confidence intervals can be found in the supplemental material as Excel tables.

This, in conjunction with the Transwell assay results, demonstrates that Na4HB at concentrations up to 10 times higher than the peri-implant level does not increase the migratory potential of the human breast cancer or mammary epithelial cell types tested.

#### 4HB does not increase the colony formation ability of cancer or epithelial cells

The effect of varying concentrations of Na4HB on the colony formation ability of human breast cancer and mammary epithelial cell types was assessed by colony count using a soft agar assay, as illustrated in the schematics of the study design (Fig. 1).

**Fig. 8:**
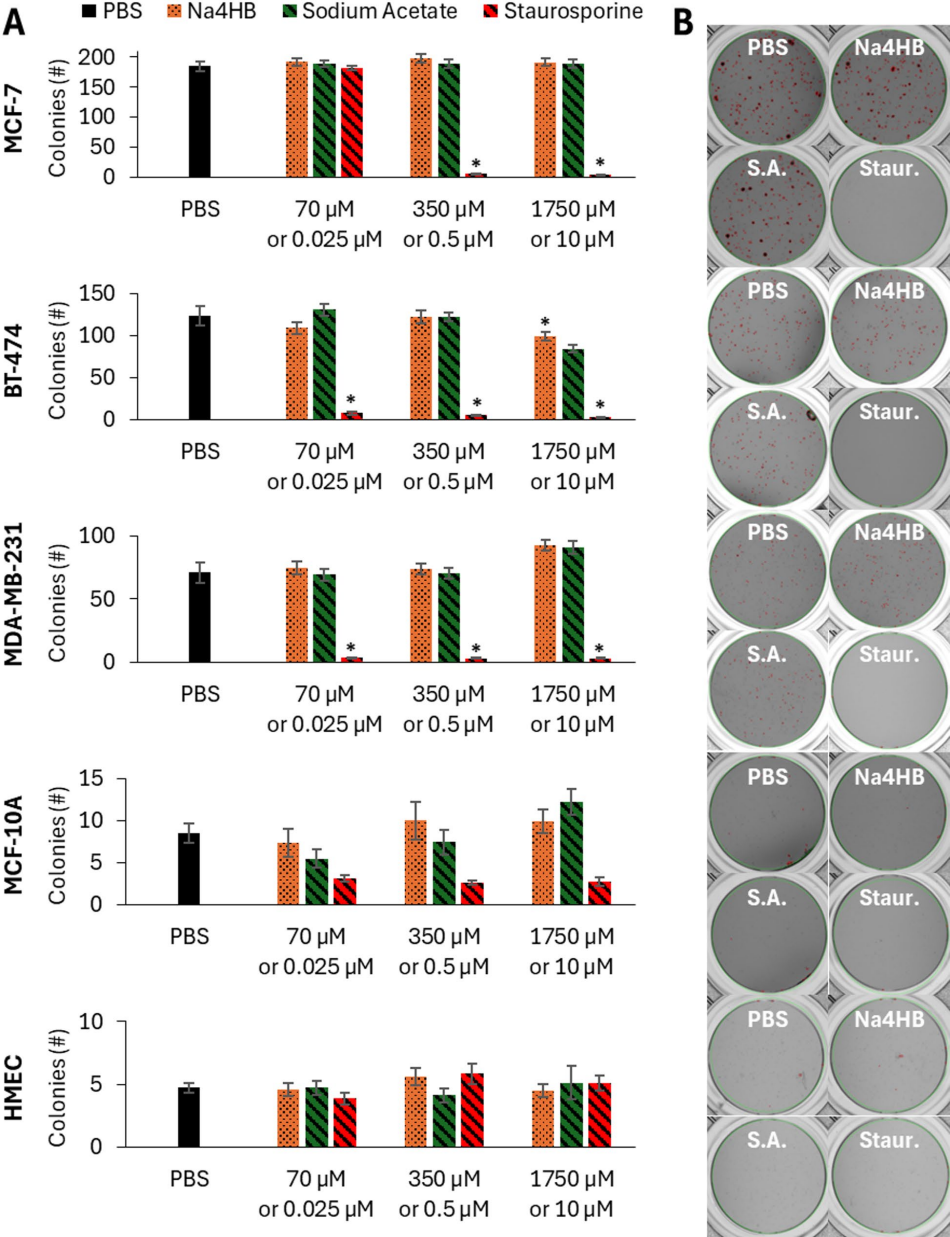
**A** Colony counts for cancer and epithelial cells after 2 weeks exposure to 70, 350, or 1750 µM Na4HB, sodium acetate (S.A.) or 0.025, 0.5, 10 µM staurosporine, measured by the soft agar assay. Data represents the average response ± standard error of the mean across 3 biological replicates. Technical replicates: MCF-7 (*n* = 32), MDA-MB-231 (*n* = 11), MCF-10A (*n* = 17), HMEC (*n* = 22). **B** Representative well plate images at the highest treatment concentrations after 14 days. Red circles indicate objects counted as colonies by the GelCount Instrument. * *p* < 0.05 to PBS

In each experiment, the PBS controls exhibited strong colony formation for each cancer cell line. In contrast, the reference compound, staurosporine, demonstrated an inhibition of colony formation across all three cell lines, indicating its consistent effectiveness as a control and the successful execution of the assay. Representative images of single wells for PBS or the highest tested concentration of Na4HB and staurosporine are shown in Fig. 8B, for each cell type. The red circles indicate colonies between 50 and 300 μm diameter that were identified and counted by the GelCount Mammalian Cell Colony Count system and software.

Prior to counting the colonies, the images were reviewed to identify invalid wells in which clonal growth of cells on the bottom surface of the plate occurred. Since overgrowth does not represent anchorage independent colony formation and interferes with the colony count image analysis, these wells were not included in the data analysis. The colony count data from the valid wells was expressed as the mean ± the standard error from the mean (SEM) across the independent experiments. No significant differences in the colony formation of BT-474, MCF-7 and MDA-MB-231 breast cancer cells were observed between Na4HB and sodium acetate or PBS at any concentration with the exception of BT-474 at the highest dose, which showed a statistically significant reduction in number of colonies formed relative to the PBS control. See comparative bar charts in Figs. 8A for each cell type including the sample size (n-value) of valid wells for each. Details on the statistical analysis results including p-values and confidence intervals can be found in the supplemental material as Excel tables.

No significant differences were observed between Na4HB and sodium acetate or PBS at any concentration with the exception of BT-474 at the highest dose, for which the number of colonies formed was significantly lower than PBS. This demonstrates that Na4HB concentrations up to 10 times higher than the peri-implant level does not increase colony formation ability of the human breast cancer or mammary epithelial cell types tested.

## Discussion

The present study aimed to provide a rigorous evaluation of the oncologic safety of P4HB scaffolds through in vitro models that assessed several key cancer hallmarks in multiple, relevant human breast cancer cell lines. The results did not reveal evidence that 4HB treatment increases the metabolic activity, cell proliferation, migration or colony forming ability of the human breast cancer or epithelial cells tested compared to controls.

Given the increasing use of P4HB scaffolds in cancer patients [4, 5], it was important to understand if the degradation of a P4HB scaffold could contribute to tumorigenesis and cancer progression, which results from the acquisition of key cancer hallmarks such as increased cell metabolism, proliferation, migratory potential and anchorage independent growth [27]. Therefore, a selection of in vitro assays based on evaluating these phenotypic attributes was used to assess the direct effect of 4HB on cancer cells [20–24]. The assays were chosen due to their mechanistic association between the aforementioned cancerous hallmarks and selected in vitro study endpoints. The utility of the MTT reagent in detecting an increase in cell metabolism is linked to enzymatic activity of reductases within the glycolytic pathway. The chromogenic transition driven by this redox mediated event allowed for a colorimetric-based measurement of cellular energetics. The increased proliferative capacity that is characteristic of cancerous cells was measured via the thymidine analog BrdU, in which high specificity antibodies were used to detect analog incorporation rates within newly synthesized DNA. The Transwell and Scratch assays were well-suited for an investigation of the cell migration phenotype due to their ability to enumerate cells that traversed a migratory barrier within an in vitro chamber. The colony formation hallmark was manifested as an anchorage-independent colony frequency count within the three-dimensional nutrient medium of the soft agar colony formation assay.

The study assessed these attributes in the context of multiple commercially available human breast cancer cell lines in order to observe the direct effects of 4HB on cellular phenotypes. The inclusion of each cell line was based on its phenotypic characteristics, relevance in human breast cancer, and the prevalence of these tumor types in breast cancer patients. Two mammary epithelial cell types were also included in the study to assess the effects of Na4HB on non-cancerous cells. This approach provided insight into whether exposure to P4HB degradation products could induce cancer-like behaviors in normal or non-malignant breast cells.

The findings of this study demonstrate that none of the cancer hallmarks evaluated, which contribute to tumorigenesis and cancer progression, were exacerbated for any of the relevant breast cancer cell lines after exposure to 4HB. The results showed that exposure to 4HB did not elicit cancerous phenotypes for mammary epithelial cells. 4HB is a natural metabolite typically present in mammalian tissue at 0.29 to 4.6 µg/g of wet tissue [28]. Importantly, the concentrations of 4HB in the peri-implant tissue of explanted (8.6 µg/g) and pre-degraded (20.5 µg/g) P4HB scaffolds are several folds higher than native tissue. However, the concentration of 4HB in the surrounding tissue of explanted P4HB was similar to that found with control polypropylene meshes (7 µg/g). An increase in the concentration of 4HB in the tissue surrounding a degrading P4HB mesh is expected compared to native tissue or a control mesh given the slow degradation of P4HB and release of 4HB, however, the values observed suggest that the 4HB does not accumulate in the tissue, but rather achieves a local concentration in the range of 8–20 µg/g (Table 2). By including both an intact P4HB scaffold and pre-degraded P4HB mesh with a lower starting Mw, the samples used effectively represent P4HB scaffolds that have been implanted for up to approximately 1 year, during which time the polymer has undergone substantial degradation (85% Mw loss and approximately 20–30% mass loss).

The tissue concentrations (µg per g wet tissue) of 4HB found in the peri-implant tissues were converted into solution concentrations (µM) by assuming the density of the tissue was 1 g/ml and using the formula weight of 126 g/mol for the sodium salt of 4-hydroxybutyrate, which is the most likely form of monomer. Thus, the highest tissue concentration of Na4HB found (20.5 µg/g wet tissue) translates to 0.02 g Na4HB/L, or approx. 163 µM. Despite exposing cells to 10 times this concentration (i.e. 1750 µM), there was no significant increase in metabolic activity, proliferation, migration, or colony formation compared to PBS or sodium acetate controls.

The results of this in vitro study support the existing clinical literature on P4HB scaffold use in breast reconstruction. Ten articles reported use of a poly-4-hydroxybutyrate (P4HB) scaffold in cancer patients undergoing breast reconstruction (9 studies) or cosmetic breast surgery (1 study) [4, 5, 7, 29–36]. Most of these studies were not designed to test the performance or safety of the P4HB material in cancer context, and most studies did not report on cancer related outcomes including cancer recurrence or metastasis rates. Thus, the current clinical literature reflects an absence of evidence rather than evidence of absence regarding oncologic risk. However, two studies did report outcomes related to cancer recurrence or malignancy [31, 35]. Sinclair (2024) reported no oncologic issues in 248 patients undergoing aesthetic breast surgery with P4HB scaffold with a mean 2.9 year follow-up [35]. Rehnke (2024) reported that no patients developed a cancer following prophylactic mastectomy and reconstruction, whereas five breasts from the therapeutic mastectomy/mesh fat graft reconstructions (9.8%) developed a local recurrence during a mean follow-up of 4.5 years [31], which is consistent with reported recurrence rates without P4HB scaffold [37, 38]. Of note, none of the five local/regional recurrence cases had radiation before recurrence, and four of the five refused any adjuvant therapies. Rehnke (2024) concluded that the results with P4HB (5.7% metastatic disease) were favorable compared to the author’s previous experiences with reconstruction with silicone (9.4% metastatic disease) [31]. While this existing clinical evidence suggests the safety of P4HB scaffolds in proximity to cancer, they represent limited evidence. Further prospective studies including comparative studies with defined oncologic endpoints are necessary to establish the long-term oncologic safety of P4HB scaffolds used in breast reconstruction more definitively.

The use of well-understood and standardized benchtop assays, like those evaluated in the present report, to quantify cellular responses adds to the evaluation of P4HB that has already been conducted. However, a limitation of this study is its focus on in vitro assays, which cannot replicate the complex biological response of the body to a foreign implant, especially in patients with a history of breast cancer. Clinically, the local tissue microenvironment can be influenced by the surgical procedure and any supportive materials used. Following the surgery, many biological processes are occurring simultaneously, including but not limited to, wound healing, tissue remodeling, hypoxia, and inflammation. All these pathways individually, and in combination, can affect the behavior of residual cancer cells. To simulate these, additional in vitro studies using co-culture systems with immune and/or tissue remodeling cells in the presence of P4HB could reveal insights into behavior of cancer cells. Moreover, future studies in more complex systems, such as orthotopic in vivo models, could further assess the oncologic safety of P4HB scaffolds in the context of a dynamic tissue microenvironment and clinical studies could provide necessary long-term and systemic response data of P4HB scaffolds in the context of breast cancer.

## Conclusion

This comprehensive, controlled in vitro evaluation suggests that 4HB does not exacerbate the cancerous properties/phenotypes of human breast cancer cell lines, and does not promote changes in cellular behavior consistent with malignant transformation of normal mammary epithelial cells tested at concentrations 10X higher than those observed in peri-implant tissue surrounding degrading P4HB scaffolds. Na4HB treatment did not increase the metabolic activity, cell proliferation, migration or colony forming ability of the breast cancer or epithelial cells tested at the concentration range evaluated (70–1750 µM). While these findings suggest that the 4HB released during the degradation of a P4HB scaffold does not promote oncogenic behavior in vitro, only a subset of cancer-related phenotypes was assessed, the assays lacked stromal and immune components, and long-term or systemic effects remain uncharacterized. These limitations underscore the need for further evaluations such as in vitro co-culture systems, in vivo studies and long-term clinical data to fully assess the oncologic safety of P4HB scaffold use in breast reconstruction. Given the continued use and the perceived benefits of supportive scaffolds in breast procedures, additional well-designed in vitro, in vivo and clinical studies will help to further support their oncological safety.

## Supplementary Information

Below is the link to the electronic supplementary material.

Supplementary Material 1

Supplementary Material 2

Supplementary Material 3

Supplementary Material 4

Supplementary Material 5

Supplementary Material 6

## Data Availability

The datasets used and analyzed during the current study are available from the corresponding author on reasonable request.

## References

[1] American Society of Plastic Surgeons. 2024 Plastic Surgery Statistics Report [Internet]. 2024. Available from: https://www.plasticsurgery.org/documents/news/statistics/2024/plastic-surgery-statistics-report-2024.pdf

[2] Jonczyk M, Jean J, Graham R, Chatterjee A. Surgical trends in breast cancer: a rise in novel operative treatment options over a 12 year analysis. Breast Cancer Res Treat. 2019;173(2):267–74. 30361873 10.1007/s10549-018-5018-1 PMC6486837

[3] Graziano FD, Plotsker EL, Rubenstein RN, Haglich K, Stern CS, Matros E, et al. National trends in acellular dermal matrix utilization in immediate breast reconstruction. Plast Reconstr Surg. 2024;153(1):E25. 10.1097/PRS.0000000000010575 PMC11305089 37092982

[4] Levy AS, Bernstein JL, Xia JJ, Otterburn DM. Poly-4-Hydroxybutyric acid mesh compares favorably with acellular dermal matrix in tissue Expander–Based breast reconstruction. Ann Plast Surg. 2020;85(S1):S2–7. 32243319 10.1097/SAP.0000000000002339

[5] Movassaghi K, Gilson A, Stewart CN, Cusic J, Movassaghi A. Prepectoral two-stage implant-based breast reconstruction with Poly-4-Hydroxybutyrate for pocket control without the use of acellular dermal matrix: a 4-year review. Plast Reconstr Surg. 2024;154(1):15. 37410610 10.1097/PRS.0000000000010914

[6] Van Natta BW, Pineda Molina C, Antonelli V, Hussey GS, Badylak SF. Histomorphologic outcomes of GalaFLEX scaffold used in breast surgery: clinical Follow-up from 6 weeks to 63 months. Aesthetic Surg J. 2025. 10.1093/asj/sjaf100 PMC12461751 40614234

[7] Bai J, Ferenz S, Fracol M, Kim JY. Revision breast reconstruction with biologic or synthetic mesh: an analysis of postoperative capsular contracture rates. Aesthetic Surg J Open Forum. 2024;6. 10.1093/asjof/ojae035 PMC11160324 38854738

[8] Wolf MT, Ganguly S, Wang TL, Anderson CW, Sadtler K, Narain R, et al. A biologic scaffold–associated type 2 immune microenvironment inhibits tumor formation and synergizes with checkpoint immunotherapy. Sci Transl Med. 2019;11:477. 10.1126/scitranslmed.aat7973 PMC7254933 30700576

[9] Williams SF, Martin DP, Moses AC. The history of GalaFLEX P4HB scaffold. Aesthetic Surg J. 2016;(Suppl):S33–42. 10.1093/asj/sjw141 PMC5070449 27697885

[10] Mamelak M. Alzheimer’ s disease, oxidative stress and gammahydroxybutyrate. Neurobiol Aging. 2007;28(9):1340–60. 16837107 10.1016/j.neurobiolaging.2006.06.008

[11] Wendt K, Patte-Mensah U-L, Eckert S, et al. Gamma-hydroxybutyrate, acting through an anti-apoptotic mechanism, protects native and amyloid-precursor-protein-transfected neuroblastoma cells against oxidative stress-induced death. Neuroscience. 2014;263:203–15. 24456637 10.1016/j.neuroscience.2013.12.067

[12] Gibson K, Nyhan W. Metabolism of [U-14 C]-4-hydroxybutyric acid to intermediates of the Tricarboxylic acid cycle in extracts of rat liver and kidney mitochondria. Eur J Drug Metab Pharmacokinet. 1989;14(1):61–70. 2759135 10.1007/BF03190843

[13] Pineda Molina C, Hussey GS, Eriksson J, Shulock MA, Cárdenas Bonilla LL, Giglio RM, et al. 4-Hydroxybutyrate promotes endogenous antimicrobial peptide expression in macrophages. Tissue Eng Part A. 2019;25(9–10):693–706. 30982430 10.1089/ten.TEA.2018.0377

[14] Zare-Zardini H, Saberian E, Jenča A, Ghanipour-Meybodi R, Jenča A, Petrášová A et al. From defense to offense: antimicrobial peptides as promising therapeutics for cancer. Front Oncol. 2024;14. 10.3389/fonc.2024.1463088 PMC11496142 39445062

[15] Huang J, Chen X, Lin H, Chen X. β-hydroxybutyrate impairs nasopharyngeal carcinoma cell aggressiveness via histone deacetylase 4 Inhibition. Mol Cell Toxicol. 2024;20(3):629–40.

[16] Mao T, Qin F, Zhang M, Li J, Li J, Lai M. Elevated serum β-hydroxybutyrate, a circulating ketone metabolite, accelerates colorectal cancer proliferation and metastasis via ACAT1. Oncogene. 2023;42(23):1889–99. 37185457 10.1038/s41388-023-02700-y

[17] Deeken CR, Matthews BD. Characterization of the mechanical Strength, resorption Properties, and histologic characteristics of a fully absorbable material (Poly-4-hydroxybutyrate—PHASIX Mesh) in a Porcine model of hernia repair. Report No.: edsbas; 2013 May. 10.1155/2013/238067 PMC3679684 23781348

[18] Martin DP, Badhwar A, Shah DV, Rizk S, Eldridge SN, Gagne DH, et al. Characterization of poly-4-hydroxybutyrate mesh for hernia repair applications. J Surg Res. 2013;184(2):766–73. 23582230 10.1016/j.jss.2013.03.044

[19] Pineda Molina, Giglio, Gandhi, Sicari, Londono H, et al. Comparison of the host macrophage response to synthetic and biologic surgical meshes used for ventral hernia repair. J Immunol Regen Med. 2019;3:13–25.

[20] Mosmann T. Rapid colorimetric assay for cellular growth and survival: application to proliferation and cytotoxicity assays. J Immunol Methods. 1983;65(1–2):55–63. 6606682 10.1016/0022-1759(83)90303-4

[21] Muir D, Varon S, Manthorpe M. An enzyme-linked immunosorbent assay for bromodeoxyuridine incorporation using fixed microcultures. Anal Biochem. 1990;185(2):377–82. 2339793 10.1016/0003-2697(90)90310-6

[22] Boyden S. The chemotactic effect of mixtures of antibody and antigen on polymorphonuclear leucocytes. J Experimental Med the. 1962;115(3):453–66. 10.1084/jem.115.3.453 PMC2137509 13872176

[23] Kramer N, Walzl A, Unger C, Rosner M, Krupitza G, Hengstschläger M, et al. In vitro cell migration and invasion assays. Mutat Res. 2013;752(1):10–24. 22940039 10.1016/j.mrrev.2012.08.001

[24] Borowicz S, Van Scoyk M, Avasarala S, Karuppusamy Rathinam MK, Tauler J, Bikkavilli RK et al. The soft agar colony formation assay. J Vis Exp. 2014;(92):E51998. 10.3791/51998 PMC4353381 25408172

[25] International Council for Harmonisation (ICH), U.S. Food and drug administration (FDA). ICH S6 (R1) addendum: preclinical safety evaluation of biotechnology-derived pharmaceuticals part II, Sect. 3.1. 2012; Available from: https://www.fda.gov/files/drugs/published/S6-(R1-Addendum--Preclinical-Safety-Evaluation-of-Biotechnology---Derived-Pharmaceuticals.pdf

[26] Pérez-Yépez EA, Ayala-Sumuano J-T, Reveles-Espinoza AM, Meza I, Toillon R-A. Selection of a MCF-7 breast cancer cell subpopulation with high sensitivity to IL-1β: characterization of and correlation between morphological and molecular changes leading to increased invasiveness. Int J Breast Cancer. 2012;2012. 10.1155/2012/609148 PMC3357940 22655200

[27] Hanahan D, Weinberg RA. Hallmarks of cancer: the next generation. Cell. 2011;144(5):646–74. 21376230 10.1016/j.cell.2011.02.013

[28] Nelson T, Kaufman E, Kline J, Sokoloff L. The extraneural distribution of γ-Hydroxybutyrate. J Neurochem. 1981;37(5):1345–8. 7299403 10.1111/j.1471-4159.1981.tb04689.x

[29] Adams WP, Baxter R, Glicksman C, Mast BA, Tantillo M, Van Natta BW. The use of Poly-4-Hydroxybutyrate (P4HB) scaffold in the ptotic breast: a multicenter clinical study. Aesthetic Surg J. 2018;38(5):502–18. 10.1093/asj/sjy022 29401215

[30] Chen Y, Wang ML, Black GG, Bernstein JL, Chinta M, Otterburn DM. Timeline and incidence of postoperative complications in prepectoral, dual-plane, and total submuscular alloplastic reconstruction with and without biosynthetic scaffold usage. Ann Plast Surg. 2023;90(6S):S466–71. 36880719 10.1097/SAP.0000000000003482

[31] Rehnke RD, Clarke JM, Goodrum AJ, Badylak SF. Absorbable biosynthetic scaffolds in place of silicone for breast reconstruction: a 9-Year experience with 53 patients. Plast Reconstr Surg Glob Open. 2024;12(5):E5821. 38798934 10.1097/GOX.0000000000005821 PMC11124690

[32] Ferenz S, Bai J, Fracol M, Kim JYS. A comparison of capsular contracture rates after immediate Implant-based breast reconstruction using biologic versus synthetic mesh. Plast Reconstr Surg Glob Open. 2024;12(8):E6031. 39157709 10.1097/GOX.0000000000006031 PMC11326475

[33] Rehnke RD, Schusterman MA, Clarke JM, Price BC, Waheed U, Debski RE, et al. Breast reconstruction using a three-dimensional absorbable mesh scaffold and autologous fat grafting: a composite strategy based on tissue-engineering principles. Plast Reconstr Surg. 2020;146(4):E409. 10.1097/PRS.0000000000007172 32969997

[34] Sigalove S, O’Rorke E, Maxwell GP, Gabriel A. Evaluation of the safety of a GalaFLEX-AlloDerm construct in prepectoral breast reconstruction. Plast Reconstr Surg. 2022;150:S75–81. 10.1097/PRS.0000000000009520 35943912

[35] Sinclair NR, Adams WPJ. Long-term outcomes of Poly-4-Hydroxybutyrate (P4HB) in aesthetic breast surgery. Aesthetic Surg J. 2024;44(12):1293–9. 10.1093/asj/sjae145 38963821

[36] Diffley M, Tang A, Sawar K, Al-Saghir T, Gonte MR, Hall J, et al. Comparative postoperative complications of acellular dermal matrix and mesh use in prepectoral and subpectoral one-stage direct to implant reconstruction: a retrospective cohort study. Ann Plast Surg. 2025;94(5):521–7. 39874556 10.1097/SAP.0000000000004233

[37] Bargon CA, Young-Afat DA, Ikinci M, Braakenburg A, Rakhorst HA, Mureau MAM, et al. Breast cancer recurrence after immediate and delayed postmastectomy breast reconstruction—a systematic review and meta‐analysis. Cancer. 2022;128(19):3449–69. 35894936 10.1002/cncr.34393 PMC9546326

[38] Casella D, Kaciulyte J, Resca L, Lo Torto F, Luridiana G, Restaino V, et al. Looking beyond the prepectoral breast reconstruction experience: a systematic literature review on associated oncological safety and cancer recurrence incidence. Eur J Plast Surg. 2022;45(2):223–31.

