# Supplementary material for: 4-Hydroxybutyrate (4HB) released from poly-4-hydroxybutyrate scaffolds does not impact hallmark phenotypes of cancer in malignant or non-malignant breast cells
Source: Breast Cancer Res. 2026 Feb 14;28:60. doi: 10.1186/s13058-026-02234-7 (PMC13001300; doi:10.1186/s13058-026-02234-7)
Supplement: Supplementary file 6 — Supplementary Material 6 [file 13058_2026_2234_MOESM6_ESM.docx]

Supplemental Information

# Culture media, chemicals and reagents for the *in vitro* study

Basal culture media were purchased from ATCC; Hybri-Care medium (ATCC, 46-X), DMEM (ATCC, 30-2002), EMEM (ATCC, 30-2003), DMEM:F12 (ATCC, 30-2006), Mammary Epithelial Cell Basal Medium (ATCC PCS-600-030), Mammary Epithelial Cell Growth Kit (ATCC PCS-600-040).The reference compounds staurosporine (Cat #: S1421, Lot #: S1421) and dasatinib (Cat #: S1021, Lot #: S1021108) were purchased from SelleckChem. Reference compound bosutinib was purchased from LC Laboratories (Cat #: B-1788, Lot #: BSB-104). Reference compounds were dissolved in DMSO as 10 mM stock solutions. Sodium acetate was purchased from Sigma-Aldrich (Cat #: 241245, Lot #: 1003680247) and stock solution was prepared in water and sterile filtered, as was Na4HB stock solution. CellTiter 96® Non-Radioactive Cell Proliferation Assay (MTT) was purchased from Promega (Cat #: G4000, Lot #: 000572589). BrdU Cell Proliferation Assay Kit was purchased from Sigma-Aldrich (Cat # 11647229001, Lot #: 79287800). IncuCyte® Clearview 96-Well Plates (Cat #: 4582, Lot #: 240228) were purchased from Sartorius Corporation. Collagen IV was purchased from Corning (Cat #: 354233, Lot #: 26024003). SeaPlaque Agarose was purchased from Lonza (Cat #50100).

# Analytical method for measuring low levels of 4HB in tissue samples

The 4HB was extracted from the tissue samples in several rounds using aqueous methanol (50%) and quantified by GC after conversion to a butyl ester derivative, butyl 4-chlorobutyrate. During derivatization of 4HB as described below, the 4HB is quantitatively converted to a butyl 4-chlorobutyrate derivative. This is advantageous, since this compound elutes as a sharp peak during the chromatography, unlike typical hydroxyl containing compounds, which have a tendency to tail and so can be difficult to quantitate. Briefly, 1 g portions of the explant samples were extracted with a solvent (50% methanol in water) designed to extract the 4HB monomer but not the residual P4HB polymer. The collected extracts were evaporated to dryness and washed with chloroform to remove soluble P4HB or P4HB oligomers so that the 4HB monomer could be selectively isolated and quantified. The extracts were derivatized by to the butyl 4-chlorobutyrate and assayed by GC as follows. Tissue extracts and standards of gamma butyrolactone were derivatized by butyl esterification using a butanolysis reaction developed and validated for quantification of low levels of 4HB in biological extracts prior to GC analysis. The butanolysis reagent consists of 1 part butanol and 1 part 4 M HCl in 1,4-dioxane. Diphenylmethane (2 mg/mL) is added to the reagent as an internal standard. A volume of 3 ml of butanolysis reagent was transferred to each extract in a capped glass vial and vortexed for 15 seconds. After vortexing, samples were sonicated at 60°C for 1 hour. Each vial was vortexed again for 15 seconds again after sonication. The vials were heated at 92°C overnight (16-20 hours) in a heating block. After cooling, 3 ml of purified water was added to wash away the bulk of the HCl. After allowing phase separation, the upper organic layer was injected onto the GC with FID detection. Separation was achieved using an SFB-35 column (Supelco, 30 m x 0.25 mm x 0.25 µm) and helium as the carrier gas. Chromatographic conditions are included in Supplemental Information Table S1. Prior to analyzing the extracts from the rabbit explants, this GC assay was validated for linearity, repeatability, accuracy, specificity, range, extraction efficiency and sample & instrument stability. The limits of detection and quantification were determined to be 1 µg and 2.5 µg of Na4HB, respectively, in 1 g of wet tissue.

# Characterization of Na4HB

## GC Analysis for Purity

Na4HB Purity was assayed by Gas Chromatography after conversion to a butyl 4-chlorobutyrate derivative via butanolysis reaction, as described above. This method determines the wt% of 4HB within a sample of P4HB or Na4HB using gamma-butyrolactone (GBL) as the standard. The monomer in P4HB is esterified and the repeating unit has a Formula Weight of 86 g/mol (i.e. C_4_H_6_O_2_), which is the same as GBL. Whereas the formula weight of Na4HB is 126 g/mol (NaC_4_H_7_O_3_) due to the added water and sodium. Thus, the mass of 4HB determined needs to be multiplied by 1.465 (i.e. 126/86) to calculate the mass of Na4HB in the sample. This can be converted to a value of % purity by dividing by the sample mass and multiplying by 100%. GC results are summarized in Table S1. An average purity of 96.2% was determined with a standard deviation of 0.2%. Thus, the sample was found to be of high purity and shown to be homogeneous due to the narrow standard deviation of the three sample fractions tested.

**Table S1. Summary of GC Purity Analysis of Na4HB Lot DM24-83-5**

| **Sample** | **Sample Mass (mg)** | **Measured Mass as GBL (mg)** | **Converted mass as Na4HB (mg)** | **Purity (%)** | **Avg. Purity (%)** |
| --- | --- | --- | --- | --- | --- |
| A | 10.81 | 7.08 | 10.38 | 96.01 | 96.2 +/- 0.2 |
| B | 10.45 | 6.87 | 10.06 | 96.25 |  |
| C | 9.79 | 6.44 | 9.44 | 96.41 |  |

## Na4HB Chemical Identity by FTIR

A sample of Na4HB was sent to Triclinic Labs in Lafayette, IN for analysis by FTIR. The powdered sample was placed on a diamond attenuated total reflectance (ATR) sampling accessory and absorbance was measured with a spectral range of 4000 cm−1 to 400 cm−1. Each spectrum was the result of 128 co-added scans acquired at 2 cm−1 resolution. Comparison was made to a sample of gamma hydroxybutyric acid sodium salt (a.k.a., Na4HB) from Sigma Aldrich. The spectra are shown in Figure S1. The spectrum includes the diagnostic OH, CH, and C=0 absorbance peaks for a hydroxy acid and there is excellent agreement between the sample DM24-83-5 and the standard spectra for Na4HB confirming the chemical identity of the compound.


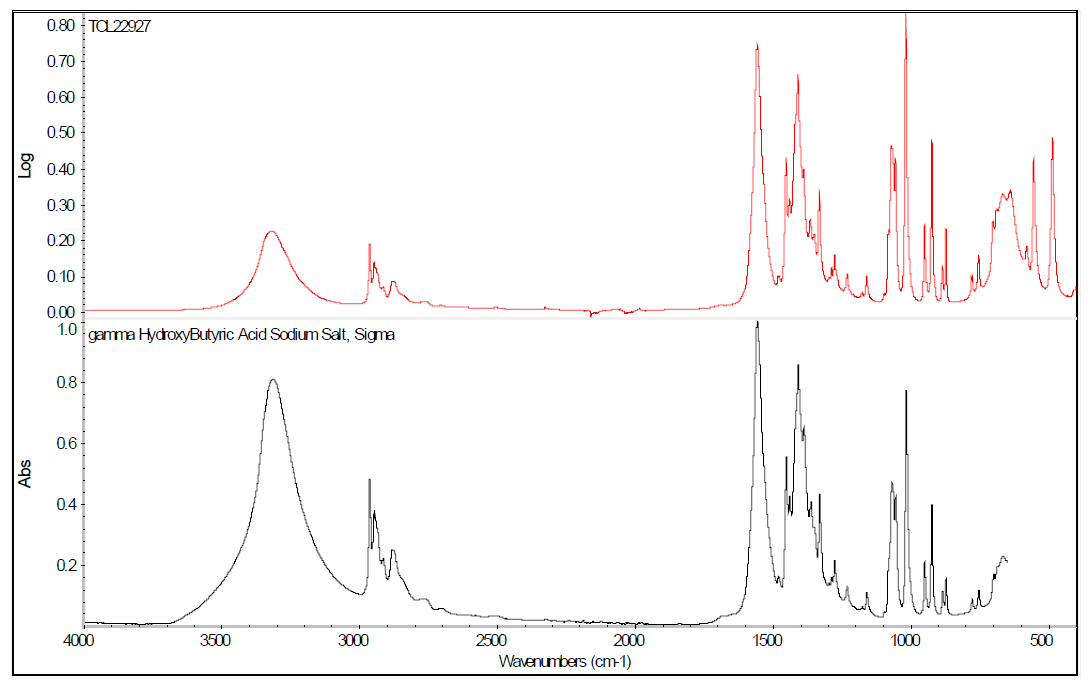


**Figure S1.** Stacked IR spectra comparing sodium 4-hydroxybutyrate, lot DM24-83-5 (TCL22927, red) and gamma hydroxybutyric acid sodium salt, Sigma (black). Data acquired at Triclinic Labs.

## Na4HB Chemical Identity by Proton NMR

The proton NMR spectrum of Na4HB (DM24-83-5) was acquired by Triclinic Labs in Lafayette, IN in deuterium oxide (approx. 11 mg/ml). The spectrum was acquired on a Bruker NEO 400 MHz spectrometer using Top-Spin GxP 4.1.4 software and the acquired ^1^H NMR spectrum was processed using TopSpin GxP 4.1.4 and referenced to the chemical shift of the residual solvent peak (e.g., D2O at 4.79 ppm). The ^1^H NMR spectrum of Na4HB lot DM24-83-5 is shown in Figure S2A. The spectrum includes the expected peaks for Na4HB at the appropriate chemical shifts, with the expected integrations and splitting patterns.. No additional peaks, such as those for gamma-butyrolactone (4.5 ppm) or P4HB oligomers (approx. 4.1 ppm) were observed. These would have been observable at about 0.2-0.5 wt% and their absence is an indication of the purity of the Na4HB preparation. Additionally, excellent agreement was found between the sample DM24-83-5 spectrum and a published spectrum (see Figure S2B).


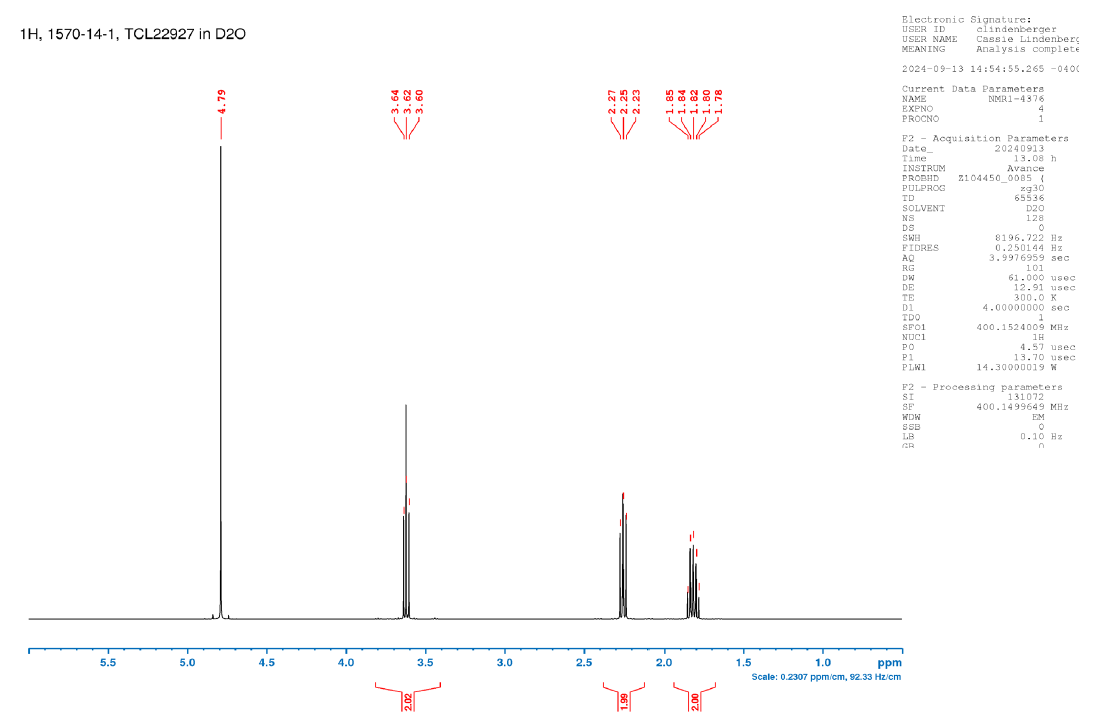


A.


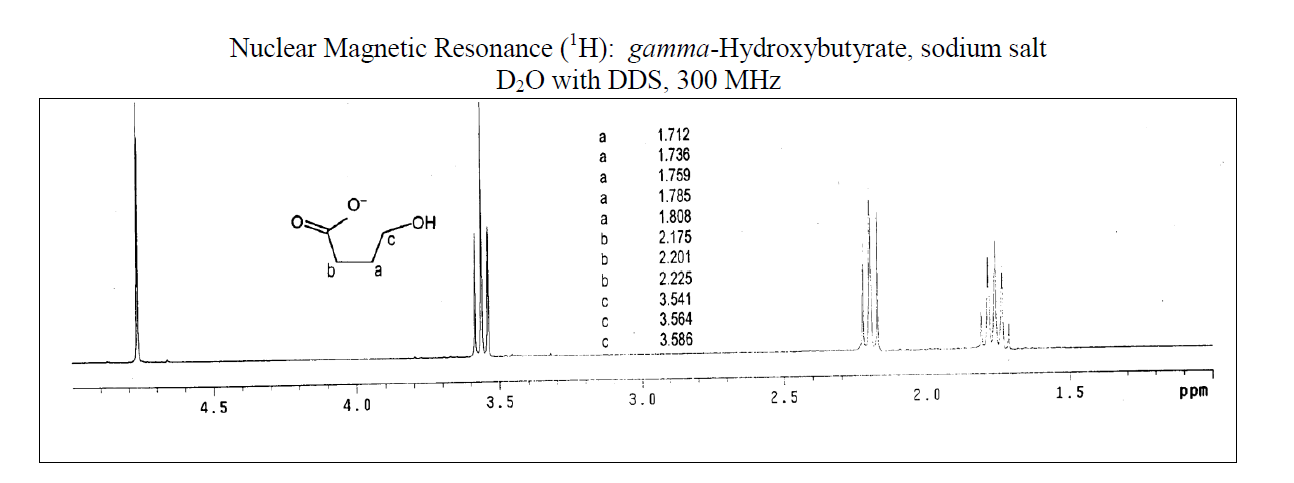


B.

**Figure S2.** A.) The ^1^H NMR spectrum of sodium 4-hydroxybutyrate (as-received, lot DM24-83-5) in deuterium oxide. Data acquired at Triclinic Labs. B.) Reference ^1^H NMR spectrum of sodium 4-hydroxybutyrate. (https://www.swgdrug.org/Monographs/GAMMA-HYDROXYBUTYRATE.pdf)

## Na4HB GC-MS Analysis for Identity

Na4HB Identity was determined by Gas Chromatography Mass Spectrometry after conversion to a di-TMS derivative. The mass spectrum is shown in Figure S3A and demonstrates the expected fragmentation pattern for the di-TMS derivative of 4HB. Additionally, excellent agreement was found between MS spectrum of DM24-83-5 and a published spectrum (see Figure S3B).


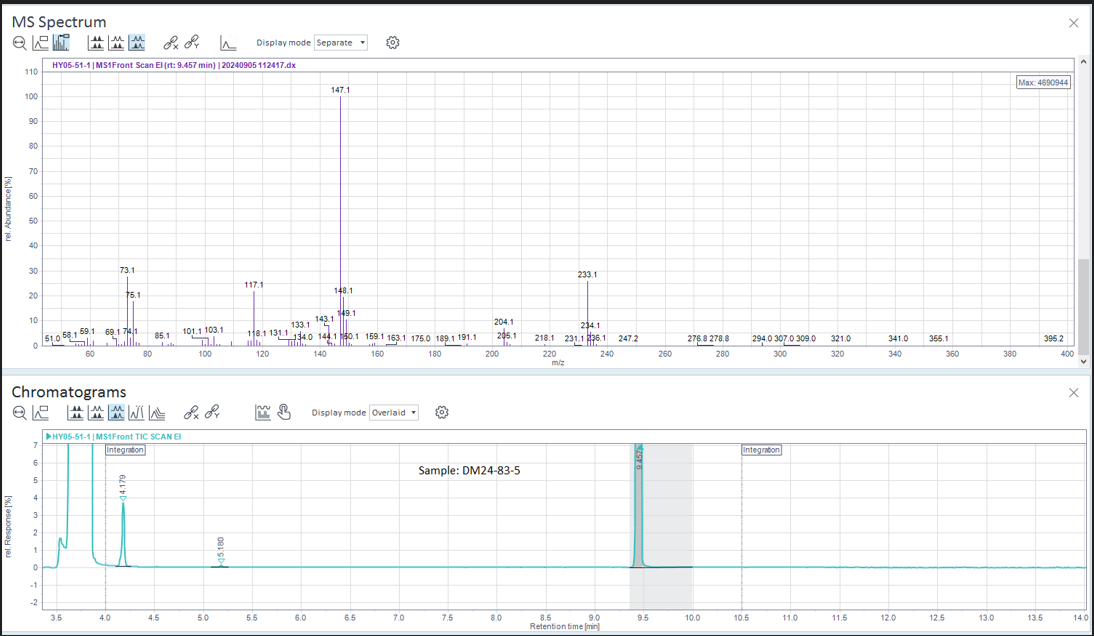


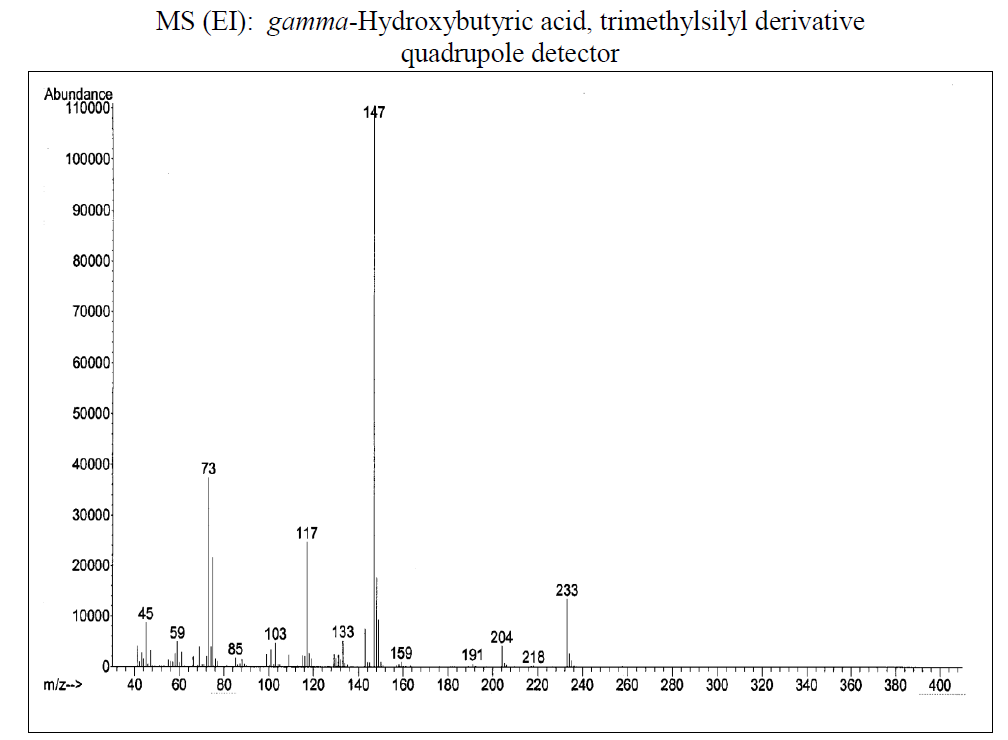


B.

**Figure S3**. A) GC-MS EI Mass Spectrum for di-TMS derivative of Na4HB, lot DM24-83-5. B) Reference EI mass spectrum for diTMS-derivative of 4-hydroxybutyrate. (<https://www.swgdrug.org/Monographs/GAMMA-HYDROXYBUTYRATE.pdf>)

## Analysis of Na4HB for Sodium and Chloride Ions and Residual Water.

The Na4HB (DM24-83-5) was assayed for sodium and chloride ion content and water by Triclinic Labs (Lafayette, IN) by Selective Ion Electrode and Karl Fischer Titration, respectively. The results are tabulated in Tables S2 and S3. The Na analysis of 17.8 wt % was very close to the expected value of 18 wt%, while the chloride analysis confirms the small amount (2.6 wt%) of residual chloride present, likely as NaCl. The small amount of water measured by KF titration (1.8 wt %) is expected for an organic salt of a short chain carboxylic acid. The presence of the small amount of residual water and chloride explain the 96.2% purity of the product. Besides chloride and water, no other contaminants were identified. During the in vitro cell assays, the 96.2% purity of the Na4HB was factored into the solutions concentrations used.

**Table S2:** Results of sodium and chloride analyses using Ion Selective Electrode for Na4HB, lot DM24-83-5.


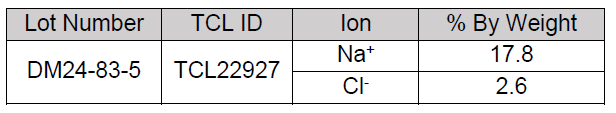


**Table S3:** Results of residual water analysis Karl Fischer Titration for Na4HB, lot DM24-83-5.


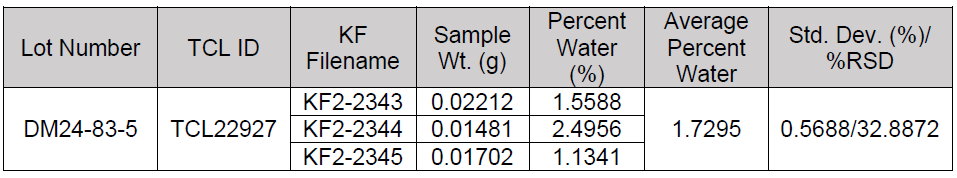


The Na4HB product isolated in this work was of high purity as measured by GC analysis (96.2 wt%). Its chemical identity was confirmed by FTIR, ^1^H-NMR and GC-MS. The expected presence of small amounts of chloride and residual water were quantified by Ion Selective Electrode and Karl Fischer Titration analyses.

## Gas Chromatograph Conditions for Analysis of the Butanolysis Derivative of 4HB

The gas chromatograph is equipped with autosampler and flame ionization detector (FID). Carrier gas is helium. The chromatographic conditions are summarized in **Table S4.**

**Table S4 GC program and conditions**

| **COLUMN** |  |
| --- | --- |
| Column | SFB-35 from Supelco, 30 m x 0.25 mm x 0.25 µm |
| Carrier gas | Helium (Grade 5.0, Ultra High Pure) |
| Flow | 2.0 mL/min (constant flow) |
| **AUTOSAMPLER** |  |
| Injection Volume | 1.0 µL |
| **INLET** |  |
| Injection Linear | Deactivated 4 mm ID split linear with Glass Wool |
| Inlet temperature | 225 °C |
| Pressure | 22.4 psi |
| Total flow | 104.8 mL/min |
| Split ratio | 50:1 |
| Split flow | 100 mL/min |
| **OVEN** |  |
| Oven temperature program | 130 °C for 2 min, 5 °C/min to 175°C, then 35 °C/min to 280°C, hold at 280 °C for 2 min |
| **Run Time** | 16 min |
| **DETECTOR** |  |
| Detection | FID |
| Detector temperature | 290 °C |
| Hydrogen (Ultra High Pure) flow | 40 mL/min |
| Air (Grade 0) flow | 450 mL/min |
| Helium makeup flow | 45 mL/min |
| Data acquisition rate | 20 Hz |

## Mw Determination of P4HB by GPC

Collagenase, Type I, from *Clostridium histolyticum* (VWR, Cat. No. 234153) was used as received. TES (2-[Tris(hydroxymethyl)-methyl-amino]-1-ethanesulfonic acid, Alfa Aesar via VWR), calcium chloride (CaCl_2_·2H_2_O, EMD via VWR) and sodium azide (NaN_3_ from Sigma) were used to prepare the TESCA buffer solution (50 mM TES, 2 mM calcium chloride, 10 mM NaN3, pH 7.4). Explanted test samples designated for Mw analysis were placed in a Falcon tube containing 25 mL collagenase (Type I) solution (1.0 mg/mL) in TESCA buffer. The tubes were incubated at 37 °C overnight (~17 hrs) at 50 rpm to digest ingrown tissue. After the incubation, any loose remaining tissue was removed manually, and the samples were rinsed with ethanol and dried. P4HB samples were dissolved at 1 mg/ml in chloroform. Weight average molecular weight analysis was performed on a EcoSEC HLC-8320GPC system with RI detector using an Agilent Technologies, PL gel, 5 µm, mixed C column (300 x 7.5 mm) with PL gel, 5 µm, 50 X 7.5mm guard column using chloroform as the eluant at 1 ml/min at 35 °C. Monodisperse polystyrene standards of known Mw were used to create the standard curve.

# Extra details on P4HB degradation

P4HB is a thermoplastic, linear polyester that can be converted into absorbable, implantable medical devices. Hydrolysis of P4HB produces the monomeric acid, 4-hydroxybutanoic acid (also known as gamma-hydroxybutyric acid or 4-hydroxybutyrate, 4HB) and its chemical structure is illustrated in **Figure S4**. When P4HB degrades in the body, the released 4HB can be further catabolized to carbon dioxide and water through the Krebs cycle. The 4HB released from a P4HB implant is an exogenous source of 4HB, since the monomer is also present naturally in mammalian tissues. 4HB is a normal constituent of the mammalian body and is found within a variety of tissues, including brain, heart, kidney, liver, lung, muscle and brown fat^[[1]](#footnote-1)^. The concentration of 4HB in these tissues ranges from 2.3 to 37.4 nmoles/g of tissue (or 0.24 to 3.8 micrograms per g of wet tissue, based on a formula weight of 104 g/mol of the 4HB free acid). Assuming soft tissue is mostly water, this translates into a concentration of approximately 3 to 40 µM in these tissues. Thus, 2.3 nmoles per g of wet tissue translates to 2.3 µmoles per L or 2.3 µM, if one assumes the tissue is mostly composed of water. A similar conversion was also applied to translate the found Na4HB tissue concentrations (µg/g) into the solution values (µM) used for the in vitro assays.


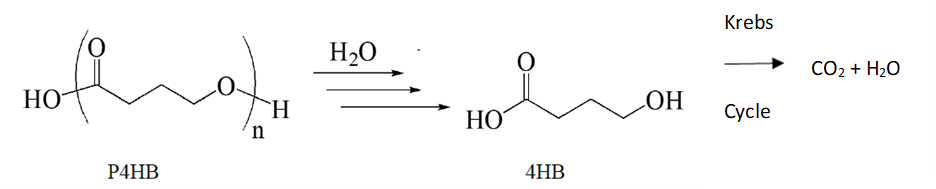


**Figure S4.** Hydrolytic degradation of poly-4-hydroxybutyrate into its constituent monomer (4HB) and eventual catabolism to carbon dioxide and water through the Krebs cycle.

# Representative Images of Migration Assays (Scratch and Transwell)

The scratch assay is a method to measure two-dimensional cell migration. A scratch wound is generated on a confluent cell monolayer, and movement tracked via microscopy or other imaging.1 The cell migration leads to the closure of the gap (wound), which is monitored by the IncuCyte® S3 Live-Cell Analysis System during the entire assay. The Incucyte® Scratch Wound Analysis Software Module allows automated detection and quantification of wound properties from each well of the 96-well plate, at a series of time points post wounding.

**Figure S5**. Representative images of gap closure (in blue color) for scratched MCF-7 cells treated with Na4HB or PBS vehicle control in the 96-well ImageLock Plate.

The Transwell migration assay involves the chemotaxis of cells through a porous membrane after the establishment of a chemoattractant gradient using two media-filled compartments. The IncuCyte Chemotaxis Cell Migration Assay approach enables automated imaging and analysis of cell migration using an optically clear membrane that allows for 96-well kinetic throughput. Cells in the IncuCyte Chemotaxis Cell Migration Assay are required to move toward a chemoattractant gradient across the membrane surface and through a pore and are recorded by phase-contrast imaging.


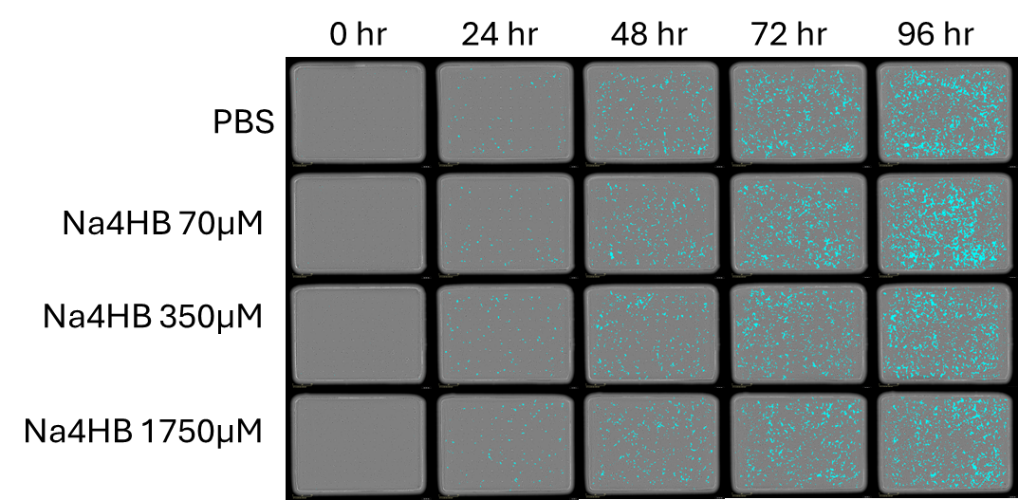


**Figure S6.** Representative of phase-contrast imaging of bottom membrane (green) for MDA-MB-231 cells in the IncuCyte Clearview 96-Well Plate.

# Soft agar assay exclusion criteria

All assays were performed on 3 independent assay days (i.e. biological replicates). The MTT, BrdU, Transwell, and scratch assays contained 6 technical replicates for each assay day/biological replicate. The mean for each assay day was calculated (n=3) and used for statistical analysis using Minitab software. The reviewer is correct, n=3 biological replicates per cell line and this clarification has been added to the text.

For the colony formation assay, technical limitations were encountered during assay development across all cell lines and within all treatment conditions. In some wells, single cells adhered to the bottom of the well and grew out as 2D colonies. These 2D outgrowths impaired the software’s ability to accurately delineate and count colonies in those wells. Therefore, we decided to exclude them from further analysis. To overcome this limitation and still perform the colony formation assay, we decided to increase the number of technical wells per assay day/biological replicate to ensure we had enough data points over the different assay days for data analysis. For final data analysis, the total number of replicates per cell line was defined by the condition that had the lowest number of valid wells (the n-value). This n-value was established to ensure that the same number of replicates were analyzed per condition. Once the n-value was determined for each cell type, the wells included in the analysis were determined by the algorithm established in the protocol. This algorithm is described below and represented graphically in Figure 1:

1. The plates were ordered sequentially by biological replicate (Bio1-A, Bio2-A, Bio3-A, Bio1-B, Bio2-B, Bio3-B, etc.).

2. The first valid well was selected from the top of the column within each condition, starting with plate Bio1-A. This was the first data point included in the analysis.

3. Counting progressed to the next biological replicate with the next valid well from the top of the column within each condition. This was the second data point included for that group.

4. This process was continued, sequentially progressing through the plate order that was established in step 1, until the n-value determined above was reached.

5. The average number of colonies formed was determined for each condition to allow for a quantitative comparison between treatments.


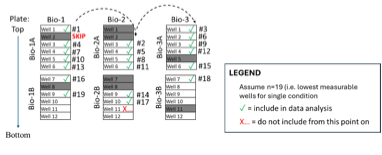


**Figure S7:** Schematic depicting the application of colony counting algorithm to a single condition. In this example, a lowest measurable n-value of 19 was used. Wells with a white background represent valid wells. Wells with a dark grey background represent invalid (i.e. wells with 2D outgrowths) that are excluded from the analysis.

1. T. Nelson, E. Kaufman, J. Kline and L. Sokoloff (1981) *J. Neurochem.* 37; 1345-1348. [↑](#footnote-ref-1)
